# TRAPS mutations in *Tnfrsf1a* decrease the responsiveness to TNFα *via* reduced cell surface expression of TNFR1

**DOI:** 10.3389/fimmu.2022.926175

**Published:** 2022-07-22

**Authors:** Takahiko Akagi, Sumie Hiramatsu-Asano, Kenta Ikeda, Hiroyasu Hirano, Shoko Tsuji, Ayano Yahagi, Masanori Iseki, Makoto Matsuyama, Tak W. Mak, Kazuhisa Nakano, Katsuhiko Ishihara, Yoshitaka Morita, Tomoyuki Mukai

**Affiliations:** ^1^ Department of Rheumatology, Kawasaki Medical School, Kurashiki, Japan; ^2^ Department of Dermatology, Okayama University Graduate School of Medicine, Dentistry, and Pharmaceutical Sciences, Okayama, Japan; ^3^ Department of Immunology and Molecular Genetics, Kawasaki Medical School, Kurashiki, Japan; ^4^ Department of General Internal Medicine 1, Kawasaki Medical School, Okayama, Japan; ^5^ Division of Molecular Genetics, Shigei Medical Research Institute, Okayama, Japan; ^6^ Department of Medical Biophysics, University of Toronto, Toronto, ON, Canada

**Keywords:** tumor necrosis factor-α, TNF receptor-associated periodic syndrome, TNF receptor 1, murine model, autoinflammatory disease

## Abstract

Tumor necrosis factor (TNF) receptor-associated periodic syndrome (TRAPS) is an autoinflammatory periodic fever syndrome associated with heterozygous mutations in *TNFRSF1A*, which encodes TNF receptor type I (TNFR1). Although possible proinflammatory mechanisms have been proposed, most previous studies were performed using *in vitro* overexpression models, which could lead to undesirable inflammatory responses due to artificial overexpression. It is crucial to reproduce heterozygous mutations at physiological expression levels; however, such studies remain limited. In this study, we generated TRAPS mutant mice and analyzed their phenotypes. Three *Tnfrsf1a* mutant strains were generated by introducing T79M, G87V, or T90I mutation. T79M is a known mutation responsible for TRAPS, whereas G87V is a TRAPS mutation that we have reported, and T90I is a variant of unknown significance. Using these murine models, we investigated whether TRAPS mutations could affect the inflammatory responses *in vivo* and *in vitro*. We found that none of the mutant mice exhibited detectable inflammatory phenotypes under standard housing conditions for 1 year. Interestingly, TRAPS mutant (T79M and G87V) mice had reduced mortality rates after the administration of lipopolysaccharide (LPS) and D-galactosamine, which induce TNFα-dependent lethal hepatitis. Moreover, TRAPS mutations strongly suppressed the development of TNFα-mediated arthritis when crossed with human TNFα transgenic mice. In *in vitro* primary bone marrow-derived macrophage cultures, the T79M and G87V mutations attenuated the inflammatory responses to TNFα compared with the wild-type, whereas these mutations did not alter the responsiveness of these cells to LPS. The T90I mutant macrophages behaved similarly to wild type in response to LPS and TNFα. The TNFR1 levels were increased in whole-cell lysates of TRAPS mutant macrophages, whereas the cell surface expression of TNFR1 was significantly decreased in TRAPS mutant macrophages. Taken together, TRAPS mutations did not augment the inflammatory responses to TNFα and LPS; instead, they suppressed the response to TNFα *via* decreased cell surface expression of TNFR1. The stimulation of lymphotoxin-α, adenosine triphosphate, and norepinephrine in primary macrophages or various stimuli in murine splenocytes did not induce detectable inflammatory responses. In conclusion, TRAPS mutations suppressed responsiveness to TNFα, and TRAPS-associated inflammation is likely induced by unconfirmed disease-specific proinflammatory factors.

## Introduction

Tumor necrosis factor (TNF) receptor-associated periodic syndrome (TRAPS; OMIM #142680, formerly known as familial Hibernian fever) is an autoinflammatory disease characterized by recurrent fever, arthralgia, myalgia, skin rash, and abdominal pain (1). TRAPS is associated with germline heterozygous mutations in the *TNFRSF1A* gene, which encodes TNF receptor type I (TNFR1), also known as CD120a (2). To date, 44 missense mutations in *TNFRSF1A* have been validated as pathogenic and 61 as likely pathogenic mutations in the Infevers database (3) (https://infevers.umai-montpellier.fr/web/, Apr. 18^th^, 2022). Almost all known TRAPS-associated mutations are point mutations causing amino acid substitutions in the extracellular domain of TNFR1 (1). Although several TRAPS-associated mutations, such as T79M and C62Y, have been established as pathogenic, the mechanisms leading to recurrent inflammatory episodes remain unclear. Furthermore, there are several mutations with unknown pathogenicity (variants of unknown significance).

We recently identified a novel p.Gly87Val (G87V) mutation in *TNFRSF1A*, harbored by two TRAPS patients in one family. Additionally, we assessed the function of this mutation using *in vitro* plasmid-transfected overexpression models and compared it with the well-known T79M TRAPS mutation (4). We showed that the G87V and T79M mutations behave in a similar manner. Based on the experimental and clinical findings, we propose that the G87V mutation is a pathogenic mutation causing TRAPS (4). Although plasmid-transfected overexpression models are widely used to investigate protein functions, these models have limitations for the analysis of TRAPS mutations. First, plasmid-based overexpression induces far greater protein expression than physiological levels, resulting in undesirable inflammatory responses. Indeed, wild-type (WT)-TNFR1 overexpression itself induces spontaneous inflammatory responses without TNFα, presumably *via* cross-linking of cell surface TNFR1, which can interfere with the inflammatory response caused by TRAPS mutations. In our previous *in vitro* analyses, HEK293 cells overexpressing T79M mutant TNFR1 had decreased inflammatory responses compared with those overexpressing WT-TNFR1, represented by the nuclear factor-κB (NF-κB) promoter activity. Similar paradoxical findings were observed in previous studies (5–7). Second, overexpression models cannot completely reproduce heterozygous states expressing both WT and mutant TNFR1 at physiological levels. As TRAPS is caused by autosomal dominant inheritance, it is important to reproduce the heterozygous mutation state.

To solve these problems, Simon et al. generated TRAPS mouse models by knocking in TRAPS mutations in *Tnfrsf1a via* homologous recombination (8). Their study significantly improved our understanding of the TRAPS pathophysiology. They showed that mutant TNFR1 accumulates intracellularly, activates mitogen-activated protein kinase (MAPK), and mediates the secretion of inflammatory cytokines upon lipopolysaccharide (LPS) stimulation. Additionally, heterozygous mutant mice were hypersensitive to LPS-induced septic shock. Based on these findings, they proposed that heterozygous mice were susceptible to inflammatory stimuli *via* a predisposed inflammatory status due to TRAPS mutations. Nonetheless, the proposed mechanisms require further validation and detailed description using TRAPS mutant mice. Furthermore, the pathogenicity of G87V and T90I mutations also remains unclear.

We generated three mutant mice by introducing T79M, G87V, and T90I mutations in *Tnfrsf1a*. T79M is a previously described TRAPS mutation (9), whereas G87V was reported by our group (4), and T90I is considered a variant of unknown significance based on Infevers. Using these murine models, we investigated whether TRAPS-associated mutations affect inflammatory responses *in vivo* and *in vitro*.

## Materials and methods

### Reagents

The following cytokines and reagents were used in this study: recombinant murine macrophage colony-stimulating factor (M-CSF; 315-02, PeproTech, Rocky Hill, NJ, USA), recombinant murine TNFα (315-0A, PeproTech), LPS from *Escherichia coli* O111:B4 (L4391, Sigma-Aldrich, St. Louis, MO, USA), concanavalin A, *Canavalia ensiformis* (ConA; C0412, Sigma-Aldrich), recombinant murine lymphotoxin-α (LTα; MBS948127, MyBioSource, San Diego, CA, USA), adenosine triphosphate (ATP; Tlrl-atpl, *In vivo* Gen, San Diego, CA, USA), norepinephrine (ALX-550-076, Enzo Life Sciences, Faimingdale, NY, USA), Phorbol 12-myristate 13-acetate (PMA; AG-CN2-0010, AdipoGen Life Sciences, San Diego, CA, USA), ionomycin (10004974, Cayman Chemical, Ann Arbor, MI, USA), cycloheximide (CHX; 0970, Bio-Techne, Minneapolis, MN, USA), restriction enzymes CviAII, AluI (New England BioLabs, Ipswich, MA, USA), and BstPI (Takara Bio, Shiga, Japan), and thioglycolate (05601, Nissui Pharmaceutical Co., Tokyo, Japan). Anti-CD3ϵ monoclonal antibody (mAb) (45-0031-82) and anti-CD28 mAb (122004) were purchased from eBioscience (San Diego, CA, USA) and BioLegend (San Diego, CA, USA), respectively. The following primary antibodies were used for western blot analysis: anti-mouse TNFR1 (13377), p38 (8690), extracellular-signal-regulated kinase (ERK) (4695), jun N-terminal kinase (JNK) (9252), NF-κB p65 (8242), phospho-p38 Thr180/Tyr182 (4511), phospho-ERK Thr202/Tyr204 (4370), phospho-NF-κB p65 Ser536 (3033), ATF-6 (65880), IRE1α (3294; all from Cell Signaling Technology, Danvers, MA, USA), TNFR1 (AF-425-PB, R&D Systems, Minneapolis, MN, USA), TNFR2 (ab109322, Abcam, Cambridge, UK), and actin (A2066, Sigma-Aldrich). The following antibodies and isotype controls were used for flow cytometry: hamster anti-mouse TNFR1 (113005, BioLegend), hamster anti-mouse TNFR2 (113405, BioLegend), rat anti-mouse/human CD11b (101245, BioLegend), rat anti-mouse CD16/CD32 antibody (553142, BD Biosciences), allophycocyanin (APC)-conjugated Armenian Hamster IgG Isotype control (400912, BioLegend), and phycoerythrin (PE)-conjugated Armenian Hamster IgG Isotype control (400908, BioLegend). All antibodies were conjugated with fluorochrome.

### Generation of *TNFRSR1A* mutant mice

*Tnfrsf1a* T79M and G87V mutant mice were generated by Setsuro Tech (Tokushima, Japan), and *Tnfrsf1a* T90I mutant mice were generated in the animal facility at Kawasaki Medical School (Kurashiki, Japan). All mutant mice (C57BL/6N background) were generated by clustered regularly interspaced short palindromic repeats (CRISPR)/Cas9-mediated insertion of the indicated mutations in exon 3 of the TNFR1 coding sequence using genome editing by electroporation of Cas9 protein (GEEP) methods (10, 11). CRISPR RNAs (crRNAs) were designed as follows: T79M: 5′-CAGGGCGGGATACAGTCTGCAGG -3′ (proto-spacer adjacent motif: PAM), G87V: 5′-GTCTGCAGGGAGTGTGAAAAAAA-3′, and T90I: 5′-GTAATTCTGGGAAGCCGTAAAGG-3′ (**Supplementary Figures 1A**, **B**). Synthetic trans-activating CRISPR RNA (tracrRNA) and a recombinant Cas9 protein were obtained from Integrated DNA Technologies Inc. (Coralville, IA, USA). Single-strand DNAs of homologous arms at both ends were synthesized with the following sequence: T79M: 5′- GTC CGA GCC CAG GGC GGG AcA tgG TCT GCA GGG AGT GTG AAA A; G87V: TCT GCA GGG AGT GTG AAA AGg tgA CCT TTA CGG CTT CCC AGA A; and T90I: TTG CAA CTG AGA CAC TGC CTG AGG TAA TTC TGG GAA GCt aTA AAG GTG CCC TTT TCA CAC TCC CTG CAG ACT GTA TCC CGC CCT GGG CTC GGA CAG TCA C -3′. Genotypes of the mutant mice were confirmed by restriction fragment length polymorphism (RFLP) analysis and Sanger sequencing (**Supplementary Figures 1C**, **D**). Off-target candidate sequences were checked using COSMID (CRISPR search with mismatches, insertions, and/or deletions, https://crispr.bme.gatech.edu/). We confirmed that there were no off-target mutations in the top three candidate sequences for each mutant mouse.

TNFR1 knockout (KO) mice were described previously (12). TNFR1 KO mice were provided by Dr. Tak W. Mak (University of Toronto, Toronto, Canada) and maintained in our facility. All mice were housed at the Kawasaki Medical School animal facility in groups (2–5 mice per cage) and maintained at 22°C in 12 h light/12 h dark cycles with free access to water and food (MF diet, Oriental Yeast Co., Tokyo, Japan).

### *In vivo* administration of LPS and D-galactosamine

LPS and D-galactosamine were administered to induce TNFα-dependent lethal hepatitis (13). Sixteen-week-old WT, T79M, G87V, and TNFR1 KO mice were intraperitoneally injected with a combination of LPS (5 or 100 μg/kg body weight; *E. coli* O111:B4, Sigma-Aldrich) and D-galactosamine (20 or 400 mg/kg body weight; Wako, Osaka, Japan). The survival of the mice was monitored for 53 h after injection.

### TNFα-mediated arthritis model

Human TNFα-transgenic (TNFtg) mice (C57BL/6N background) were obtained from Taconic Biosciences (#1006, Hudson, NY, USA). TNFtg hemizygous mice spontaneously develop arthritis on the front and hind paws at approximately 8 weeks of age, which progresses over time (14). T79M and G87V mutant mice were crossed with TNFtg mice to generate double-mutant mice. The mice were monitored for signs of arthritis in a blinded manner, and each limb was individually scored on a scale of 0–4. Scores were assigned at the age of 17 weeks based on the extent of erythema or swelling present in each limb, assigning a maximum score of 16 per mouse, as described previously (15, 16).

### Histological analyses

G87V mutant mice were housed in a clean area at the Kawasaki Medical School animal facility until the age of 33 weeks. After euthanasia, liver, lung, spleen, and inguinal lymph node samples were collected from the mice. The organs were fixed in 4% paraformaldehyde for 2 days and then embedded in paraffin. Sections from these samples were stained with hematoxylin and eosin. Digital images of the sections were obtained using a BZX-700 microscope (Keyence, Osaka, Japan).

### Primary murine bone marrow-derived macrophage culture

Primary murine bone marrow-derived macrophage culture was performed as described previously (17). Mouse bone marrow cells were isolated from the long bones of 8- to 12-week-old mice. Non-adherent bone marrow cells were seeded at a density of 3.0×10^5^ cells/mL on 12-well plates for gene expression analyses and on 6-well plates or 60 mm dishes for protein detection. The cells were incubated for 5 days in α-minimum essential medium (MEM) including 10% fetal bovine serum (FBS) and supplemented with 25 ng/mL M-CSF at 37°C and 5% CO_2_. The bone marrow-derived macrophages were stimulated with TNFα (20 or 100 ng/mL), LPS (100 ng/mL), ATP (5 mM), LTα (100 ng/mL), or norepinephrine (10 mM). ATP was added 30 min prior to the collection of the culture supernatant.

### Splenocyte culture

Spleens were isolated from 12-week-old mice and homogenized aseptically. After the lysis of red blood cells, spleen cells were plated at a density of 2.5×10^6^ cells/mL in 12-well plates in Roswell Park Memorial Institute medium (RPMI-1640) including 10% FBS and supplemented with 5×10^-5^ mM 2-mercaptoethanol (198-15781, FUJIFILM Wako Chemicals, Osaka, Japan). The splenic cells were stimulated with LPS (100 ng/mL), TNFα (100 ng/mL), PMA (50 ng/mL), ionomycin (1 μg/mL), ConA (4 μg/mL), and immobilized anti-CD3ϵ antibody and anti-CD28 antibodies (3 μg/mL). To immobilize the anti-CD3ϵ antibody, 12-well plates were coated with 1 mL/well of 1 μg/mL anti-CD3ϵ antibody in RPMI-1640 for 2 h at 37°C. Wells were washed twice with phosphate-buffered saline (PBS) and then blocked with RPMI-1640 containing 10% FBS for 30 min at 37°C before further washing step and addition of cells. After stimulation, total RNA was extracted from cultured cells and subjected to real-time quantitative polymerase chain reaction (qPCR) analysis.

### Real-time quantitative polymerase chain reaction

qPCR was performed as previously described (18, 19). Total RNA was extracted from cultured cells using RNAiso Plus (9108, Takara Bio). Total RNA was isolated from mouse whole blood using PAXgene Blood RNA Tubes and kit (761265, BD Biosciences, San Jose, CA, USA; 762174, Qiagen, Hilden, Germany), as reported previously (20). RNA was then solubilized in ribonuclease (RNase)-free water. Complementary DNA was synthesized using the PrimeScript RT Reagent Kit (RR037, Takara Bio). qPCR was performed using TB Green PCR Master Mix (RR820, Takara Bio) with the StepOnePlus Real-Time PCR System (Thermo Fisher Scientific, Cleveland, OH, USA). Gene expression levels were calculated relative to *Hprt* expression using the ΔΔCt method and normalized to the control samples. The primers used in this study were as follows: 5′-tcctcctcagaccgctttt-3′ and 5′-cctggttcatcatcgctaatc-3′ for *Hprt*; 5′-agttgacggaccccaaaag-3′ and 5′-agctggatgctctcatcagg-3′ for *Il1b*; 5′-aacgatgatgcacttgcaga-3′ and 5′-ccagaggaaattttcaataggc-3′ for *Il6*; 5′-catcttctcaaaattcgagtgaca-3′ and 5′-tgggagtagacaaggtacaaccc-3′ for *Tnf*, 5′-tgcaagacatgtcggaaaga-3′ and 5′-caggtagcgttggaactggt-3′ for *Tnfrsf1a*; 5′-gaggcccaagggtttcag-3′ and 5′-ggcttccgtgggaagaat-3′ for *Tnfrsf1b*; and 5′-ggactctgatcatggcactg-3′ and 5′-ctgatccatgcattggtaggt-3′ for *Tlr4*; 5′-cagactacgtgcacctctgc-3′ and 5′-ctgggtccaagttgaacagaat-3′ for unspliced *Xbp1 (uXbp1);* 5′-gctgagtccgcagcaggt-3′ and 5′-ctgggtccaagttgaacagaat-3′ for spliced *Xbp1 (sXbp1)*. All qPCR reactions yielded products with single peak dissociation curves.

### Western blotting

Western blotting was performed as described (21, 22). Cultured cells were washed with ice-cold PBS and lysed with RIPA lysis buffer (R0278; Sigma-Aldrich) containing protease and phosphatase inhibitor cocktails (P8340, P5726, and P0044; Sigma-Aldrich). Protein concentrations were determined using a BCA Protein Assay Kit (23227, Thermo Fisher Scientific). All samples were resolved using sodium dodecyl sulfate-polyacrylamide gel electrophoresis and transferred onto PVDF membranes. For blocking, 5% skim milk in Tris-buffered saline with 0.1% Tween-20 (TBS-T) or 5% bovine serum albumin (BSA) in TBS-T was used for non-phosphorylated and phosphorylated proteins, respectively. After blocking, the membranes were incubated with the indicated primary antibodies, followed by incubation with appropriate horseradish peroxidase-conjugated species-specific secondary antibodies. Bands were detected using the SuperSignal West chemiluminescent substrate (Thermo Fisher Scientific) and visualized using ImageQuant LAS-4000 (GE Healthcare, Little Chalfont, UK). Actin was used as the loading control to normalize the amount of protein.

### Cycloheximide assay

Primary bone marrow-derived macrophages were prepared as described above. Macrophages were incubated with cycloheximide (CHX) (20 μg/mL) for the indicated times. Total cell lysates were collected using the RIPA buffer. Western blotting was performed as described. Signal intensities were quantified using Image Studio Lite (Ver 5.2, LI-COR Biosciences, Lincoln, NE, USA).

### Enzyme-linked immunosorbent assay

TNFα and IL-1β concentrations in the serum samples and culture supernatants were measured using sandwich enzyme-linked immunosorbent assay (ELISA) kits (R&D Systems) as reported (17). Soluble TNFR1 (sTNFR1) concentrations in the culture supernatants and blood serum were measured using sandwich ELISA kits (RayBio, Norcross, GA, USA). All the kits were used in accordance with the manufacturer’s protocol. The optical density of each well was measured at 450 nm using a microplate reader (Varioskan Flash, Thermo Fisher Scientific), and the concentrations of each sample were calculated based on standard curves.

### Flow cytometry

The expression of TNFR1 and TNFR2 on the surface of peritoneal macrophages was analyzed using a flow cytometer (FACSCanto II; BD Biosciences). Three days after intraperitoneal injection of 2 mL 3% thioglycolate (05601, Nissui), peritoneal exudate cells were collected by rinsing the abdominal cavity twice with 5 mL PBS. Contaminating red blood cells were lysed using red blood cell lysis buffer (00-4333-57; eBioscience). Single-cell suspensions were incubated with anti-mouse CD16/CD32 antibody on ice for 5 min to block Fc receptors; thereafter, the cells were stained with anti-CD11b, anti-TNFR1, and anti-TNFR2 antibodies. Adequate isotype controls were prepared for all samples. Dead cells were excluded using Zombie dyes (423113, BioLegend). All data were analyzed using the FlowJo software (version 10.7.2, BD Biosciences).

### Ethics statement

All animal experiments were approved by the Institutional Safety Committee for Recombinant DNA Experiments (18-09, 18-14, 18-15, 18-17, 19-27, 21-35) and the Institutional Animal Care and Use Committee of Kawasaki Medical School (18-099, 18-110, 18-125, 20-097, 21-110). All experimental procedures were conducted according to institutional and NIH guidelines for the humane use of animals.

### Statistical analysis

All values are presented as mean ± standard deviation (SD). A two-tailed unpaired Student’s *t*-test was used to compare two groups, and a one-way analysis of variance (ANOVA) followed by Tukey’s *post-hoc* test was used to compare three or more groups. The mortality study, following LPS challenge, was performed using the log-rank test. A *p* value lower than 0.05 was considered statistically significant. All statistical analyses were performed using GraphPad Prism 5 (GraphPad Software, San Diego, CA, USA).

## Results

### Inflammatory phenotypes were undetectable in the TRAPS mutant mice under standard housing conditions

**Figure 1:**
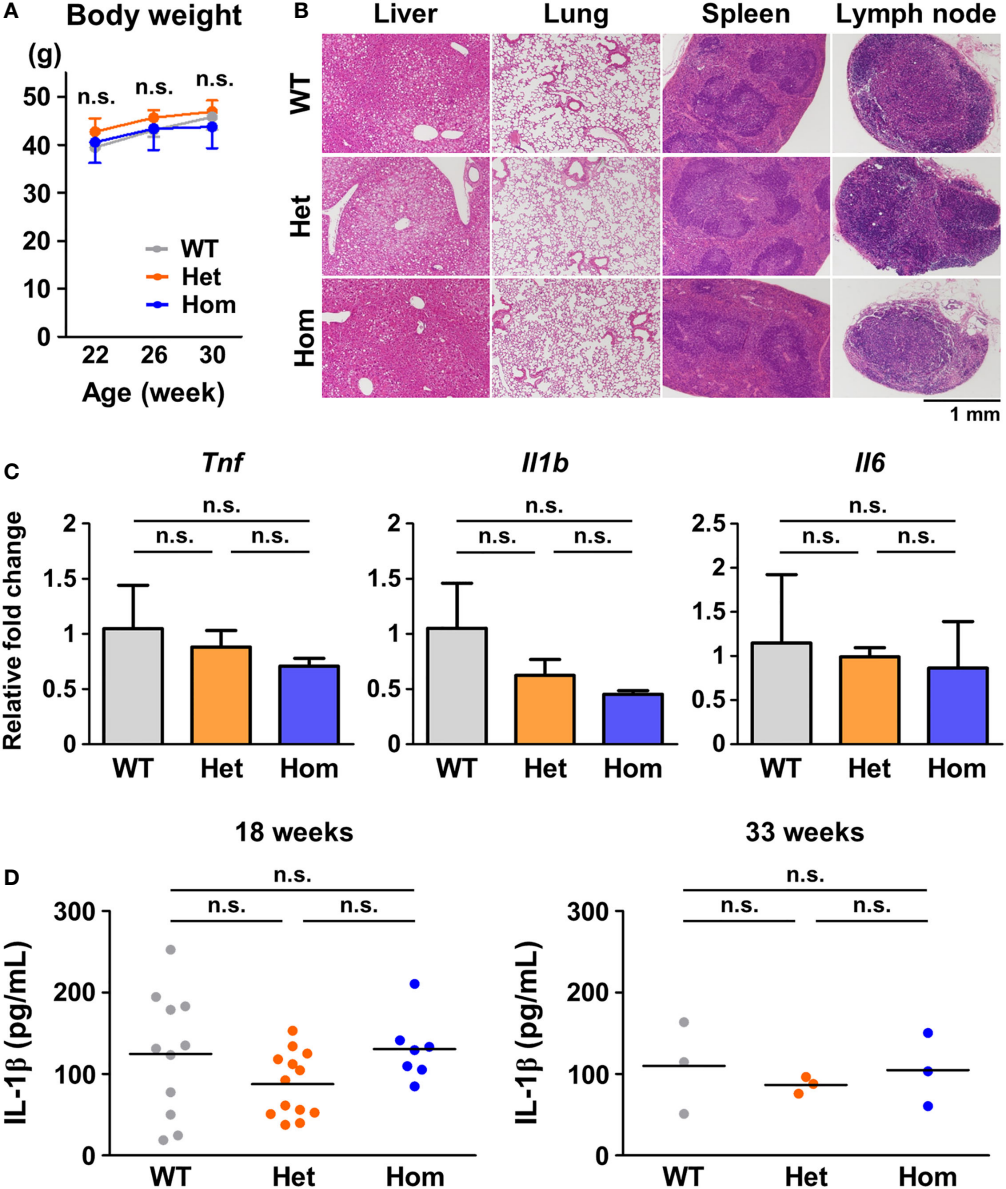
No detectable inflammatory phenotypes in G87V TRAPS mutant mice under standard housing conditions. **(A-D)** G87V TRAPS mutant mice were observed until the age of 33 weeks under standard housing conditions. **(A)** Body weights. **(B)** Representative histological images of stained tissue sections at 33 weeks of age. The liver, lung, spleen, and inguinal lymph node were stained with hematoxylin and eosin. **(C)** mRNA expression in the whole blood of the G87V mutant mice at 33 weeks of age (*n* = 3 each). Whole blood RNA samples were collected by using PAXgene RNA collection tubes. Gene expression levels relative to *Hprt* were calculated and normalized to the expression level of the WT mice. **(D)** Serum concentrations of IL-1β were determined by ELISA at 18 and 33 weeks of age. Each dot denotes an individual mouse. Values are presented as means ± standard deviation. TRAPS, Tumor necrosis factor (TNF) receptor-associated periodic syndrome; IL, interleukin; WT, wild-type; Het, heterozygote; Hom, homozygote; n.s., not significant.

To investigate the pathogenesis of TRAPS, we generated mice harboring T79M and G87V TRAPS mutations in the *Tnfrsf1a* gene using the CRISPR/Cas9 system (**Supplementary Figure 1**). We examined whether TRAPS mutant mice developed inflammatory phenotypes under standard housing conditions or in response to inflammatory stimuli. TRAPS mutant mice grew normally like WT mice without any noticeable inflammation under standard housing conditions (**Figure 1A**). Histopathological analysis of G87V mutant mice did not show any inflammatory or structural abnormalities in the liver, lung, spleen, and lymph nodes (**Figure 1B**). Moreover, the mRNA expression levels of inflammatory cytokines (*Tnf*, *Il1b*, and *Il6*) in the whole blood cells of TRAPS mutant mice were similar to those in WT mice (**Figure 1C**). Serum concentrations of IL-1β in heterozygous and homozygous mutant mice were similar to those in WT mice (**Figure 1D**). The serum concentrations of TNFα were below the detectable limit in all mice (data not shown). In addition, blood smears from TRAPS mutant mice revealed no significant changes in white blood cell counts and fractions (data not shown). Collectively, these data indicate that TRAPS mutant mice did not show any detectable inflammatory phenotypes under standard housing conditions, which is consistent with previously reported findings in T79M and C62Y TRAPS mutant mice generated by Simon et al. (8).

### Decreased lethal responses to LPS and D-galactosamine and diminished TNFα-mediated joint inflammation in TRAPS mutant mice

**Figure 2:**
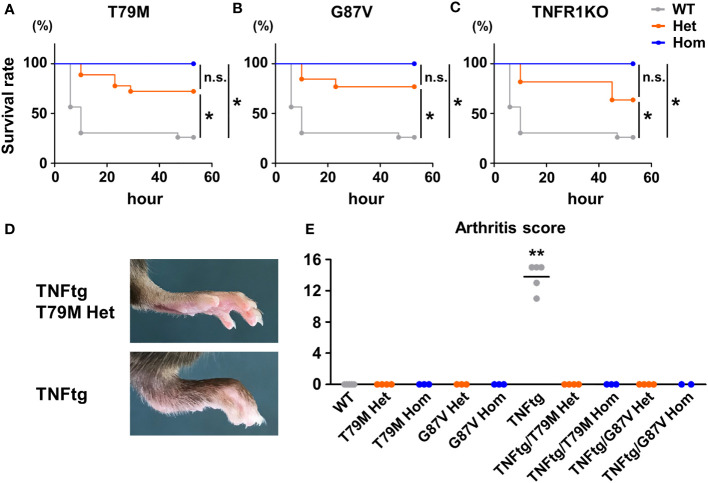
TRAPS mutations strongly suppressed lethal response by LPS and D-galactosamine and diminished TNFα-mediated arthritis. **(A-C)** T79M and G87V TRAPS mutant mice and TNFR1 KO mice were intraperitoneally administrated with LPS (100 μg/kg body weight) and D-galactosamine (400 mg/kg body weight). Survival rates of the mice after the administration of LPS and D-galactosamine. Survival rates of WT (*n* = 23), T79M strain **(A)**; Het (*n* =18), Hom (*n* = 9), G87V strain **(B)**; Het (*n* =13), Hom (*n* = 13), and TNFR1 KO strain **(C)**; Het (*n* =11), Hom (*n* = 9). Gray, orange, and blue lines indicate WT, heterozygotes, and homozygotes, respectively. *p*-values for the differences between subgroups of mice were calculated by log-rank test using stratified analysis. **(D, E)** T79M or G87V mutant mice were crossed with TNFtg mice, and the severity of arthritis was evaluated. WT (*n* = 5), T79M Het (*n* = 4), T79M Hom (*n* = 3), G87V Het (*n* = 3), G87V Hom (*n* = 3), TNFtg (*n* = 5), TNFtg/T79M Het (*n* = 4), TNFtg/T79M Hom (*n* = 3), TNFtg/G87V Het (*n* = 4), TNFtg/G87V Hom (*n* = 2). **(D)** Representative images of the hind paw of TNFtg T79M heterozygous mouse and TNFtg mouse. **(E)** Arthritis scores of the mice at the age of 17 weeks. **p<0.01 *vs*. any other groups. WT, wild-type; TNFtg, TNFα transgenic; TNFα, Tumor necrosis factor α; TRAPS, TNF receptor-associated periodic syndrome; IL, interleukin; LPS, lipopolysaccharide; TNFR1, TNF receptor type I; TNFtg, Human TNFα-transgenic mice; Het, heterozygote; Hom, homozygote. *p<0.01; n.s., not significant.

In terms of inflammatory stimuli, a previous study reported that T79M and C62Y mutant mice were susceptible to LPS and D-galactosamine stimulation (8). They showed that heterozygous mice had increased lethal responses to LPS and D-galactosamine, whereas homozygous mice were resistant to the stimulation, similar to TNFR1 KO mice (8). To confirm these findings, we initially tested the intraperitoneal administration of LPS (5 μg/kg body weight) and D-galactosamine (20 mg/kg body weight), at the doses tested previously (8). In our experimental settings, no death was observed after stimulation in all WT, T79M, and G87V mutant mice (data not shown). Next, we increased the doses of LPS and D-galactosamine to 100 μg/kg and 400 mg/kg, respectively; the doses are widely used to induce lethal hepatitis (23, 24). We found that the doses induced lethal responses in the WT mice. Notably, we found that heterozygous T79M and G87V mice had decreased lethal responses to stimulation and that homozygous T79M and G87V mice were completely tolerant to these stimuli (**Figures 2A**, **B**). The survival rates of TRAPS mutant heterozygous and homozygous mice were similar to those of TNFR1 KO heterozygous and homozygous mice, respectively (**Figure 2C**).

Since the inflammatory responses after LPS and D-galactosamine administration are predominantly mediated by TNFα (13), we next investigated whether TRAPS mutations attenuate the inflammatory response to TNFα. We generated double-mutant mice by crossing T79M or G87V mutant mice with TNFtg mice, which develop TNFα-mediated joint inflammation (14). *Tnfrsf1a*-WT TNFtg mice developed prominent joint swelling at the age of 17 weeks, whereas T79M heterozygous TNFtg and G87V heterozygous TNFtg mice did not exhibit any detectable joint swelling or deformities (**Figures 2D**, **E**). These findings indicated that T79M and G87V TRAPS mutant mice were resistant to TNFα-mediated joint inflammation.

### No significant increase in the responsiveness to LPS in TRAPS mutant macrophages

**Figure 3:**
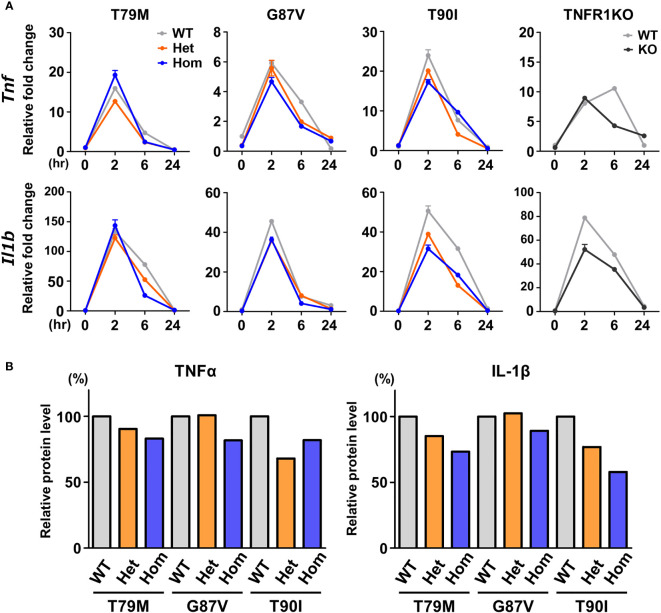
No substantial changes in the responsiveness to LPS in the TRAPS mutant macrophages. Bone marrow-derived macrophages were stimulated with LPS (100 ng/mL). After stimulation, the RNA and supernatant samples were collected at the indicated time points. Cell culture experiments were performed separately for each strain. **(A)** qPCR analysis. The mRNA expression levels of *Tnf* and *Il1b* were determined. Gray, orange, blue, and dark gray lines indicate WT, heterozygous, homozygous, and TNFR1KO mice, respectively. **(B)** Relative protein levels of TNFα and IL-1β in the culture supernatants. Culture supernatants were collected 6 h after LPS stimulation, and the concentrations of TNFα and IL-1β were determined by ELISA. Levels were calculated relative to those of the WT in each strain. TNFα, Tumor necrosis factor α; TRAPS, TNF receptor-associated periodic syndrome; IL, interleukin; LPS, lipopolysaccharide; TNFR1, TNF receptor type I; KO, knockout; qPCR, real-time quantitative polymerase chain reaction; WT, wild-type; Het, heterozygote; Hom, homozygote.

The secretion of IL-1β and TNFα was reported to be increased in T79M heterozygous macrophages compared with the expression in WT controls (8). Thus, we assessed whether TRAPS-mutant bone marrow-derived macrophages are more responsive to LPS *in vitro*. mRNA expression levels of *Tlr4*, which is the key receptor of LPS, were not altered by the *Tnfrsf1a* mutations (**Supplementary Figure 2**). Macrophages were stimulated with 100 ng/mL LPS. We found that *Tnf* and *Il1b* mRNA expression was not increased in T79M and G87V TRAPS mutant cells compared with that in WT cells (**Figure 3A**). In addition, TNFα levels in the culture supernatants were comparable, with some variations, between TRAPS mutant and WT cells (**Figure 3B**). The IL-1β levels in the culture supernatants were not elevated in TRAPS mutant cells, and were rather modestly decreased in T90I mutant cells (**Figure 3B**). As the secretion of IL-1β is triggered by inflammasome activation (25), macrophages were treated with ATP after LPS stimulation. The secretion of IL-1β was significantly enhanced by ATP, and the increased levels were comparable among the WT, T79M heterozygous, and T79M homogenous mutant mice (**Supplementary Figure 3**).

**Figure 4:**
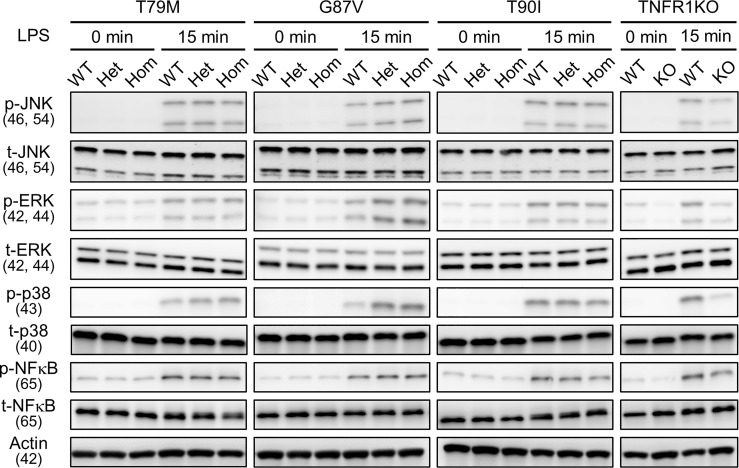
No substantial changes in LPS-induced activation of MAPK and NF-κB pathways by the TRAPS mutations. Primary murine bone marrow-derived macrophages with the indicated mutations were stimulated with 100 ng/mL LPS). Protein samples were collected before and 15 min after stimulation. Cell culture experiments were performed separately for each strain. Phospho- or total-JNK, ERK, p38, and NF-κB p65 levels were detected using specific antibodies. Actin was used as a loading control. TRAPS, TNF receptor-associated periodic syndrome; LPS, lipopolysaccharide; NF-κB, nuclear factor-κB; ERK, extracellular-signal-regulated kinase; JNK, jun N-terminal kinase; WT, wild-type; Het, heterozygote; Hom, homozygote.

Next, we investigated whether LPS affects the downstream signaling pathways. We determined the phosphorylation status of JNK, ERK, p38, and NF-κB p65 following LPS stimulation. We found that their phosphorylation patterns were not altered by *Tnfrsf1a* mutations (T79M, G87V, and T90I) (**Figure 4**). Collectively, we found that TRAPS mutant macrophages did not have increased responses to LPS stimulation compared with WT macrophages.

### Decreased responses to TNFα in TRAPS mutant cells

**Figure 5:**
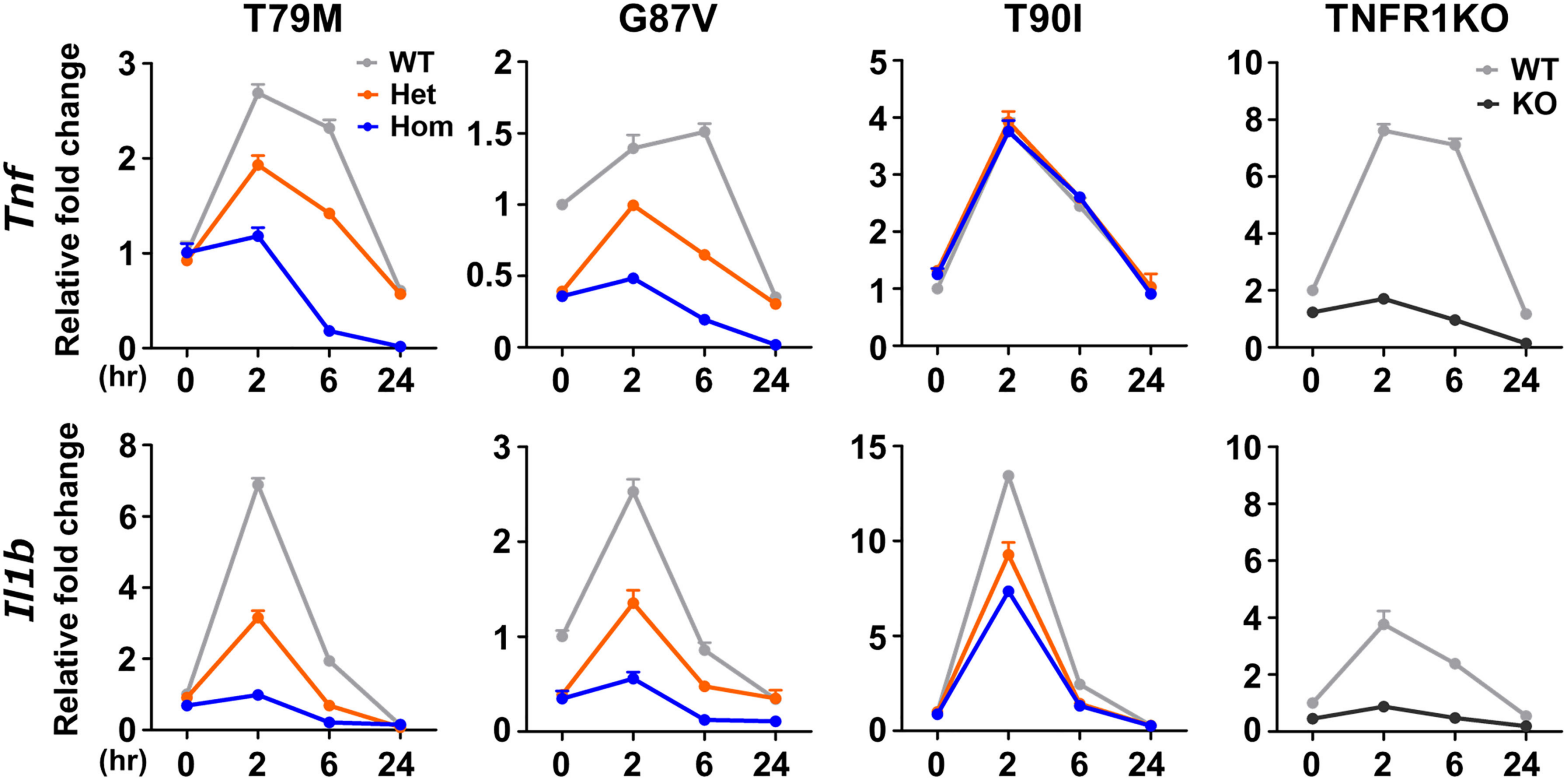
Decreased responsiveness to TNFα in the TRAPS mutations. Primary murine bone marrow-derived macrophages were stimulated with 100 ng/mL TNFα. RNA samples were collected at indicated time points. Cell culture experiments were performed separately for each strain. The mRNA expression levels of *Tnf* and *Il1b* were determined using qPCR. The WT levels at 0 h were set to 1. Gray, orange, blue, and dark gray lines indicate WT, heterozygous, homozygous, and TNFR1KO mice, respectively. TNFα, Tumor necrosis factor α; TRAPS, TNF receptor-associated periodic syndrome; WT, wild-type; Het, heterozygote; Hom, homozygote.

In a previous *in vitro* study, T79M mutant TNFR1 overexpressing cells had enhanced TNFα-mediated NF-κB activation compared with WT TNFR1-expressing cells, represented by increased phosphorylation of Ser536 in NF-κB p65 (26). Thus, we assessed the reactivity of TNFα by stimulating primary murine macrophages with 100 ng/mL TNFα. We found that *Tnf* and *Il1b* mRNA expression was the highest in WT cells, and that T79M and G87V TRAPS mutations reduced gene expression in an allele dose-dependent manner (**Figure 5**). The decreased expression levels in homozygous mutant cells were similar to those observed in TNFR1 KO cells. In contrast, T90I mutant macrophages and WT cells responded similarly to TNFα (**Figure 5**).

**Figure 6:**
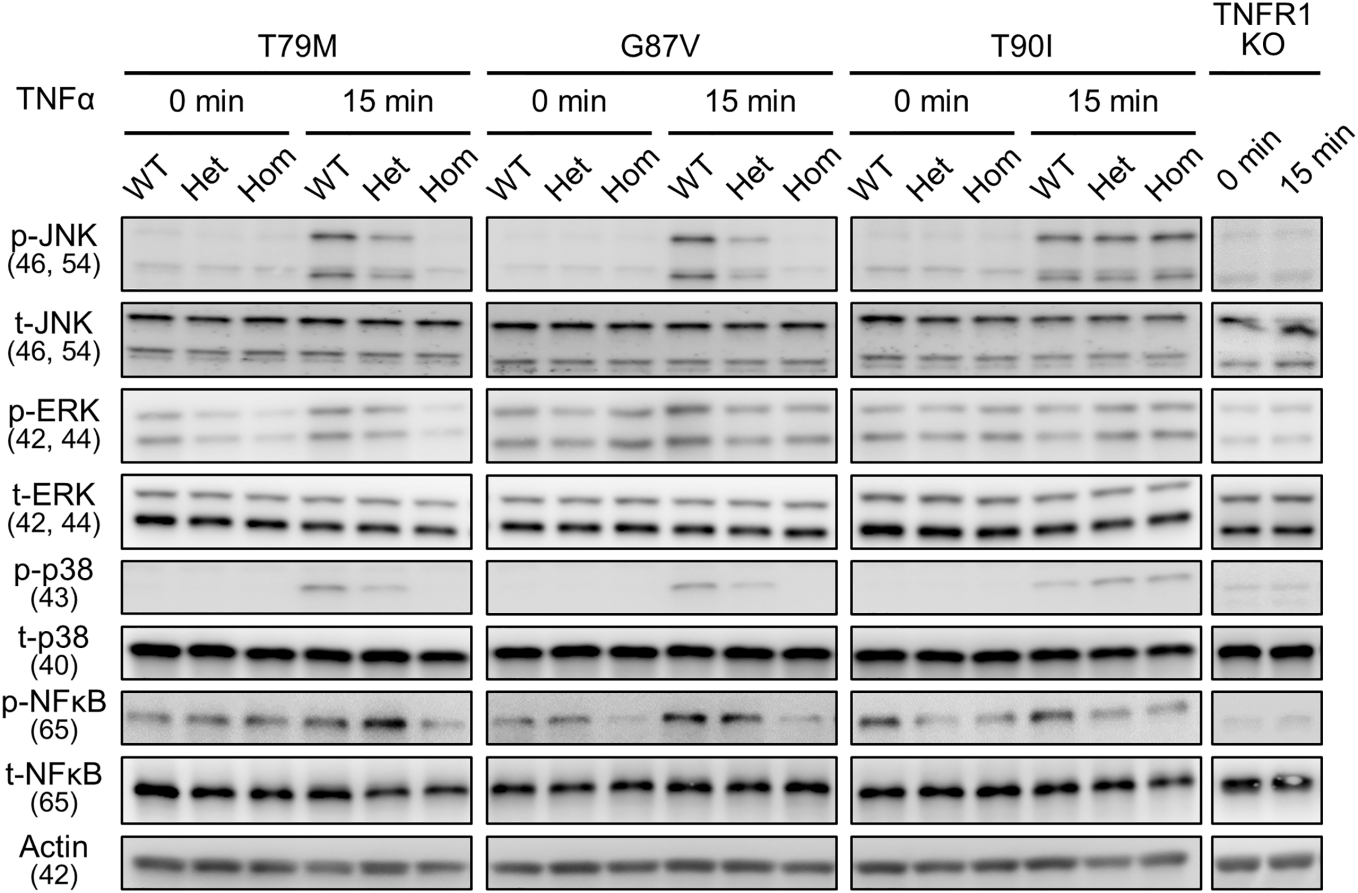
Reduced activation of MPAK and NF-κB signaling pathways in response to TNFα by the TRAPS mutations. Primary murine bone marrow-derived macrophages were stimulated with 20 ng/mL TNFα. Protein samples were collected before and 15 min after stimulation. Cell culture experiments were performed separately for each strain. Phospho- or total-JNK, ERK, p38, and NF-κB p65 levels were detected using specific antibodies. Actin was used as a loading control. TNFα, Tumor necrosis factor α; TRAPS, TNF receptor-associated periodic syndrome; NF-κB, nuclear factor-κB; WT, wild-type; Het, heterozygote; Hom, homozygote.

To determine whether downstream intracellular signaling pathways are also affected by TRAPS mutations, we evaluated the phosphorylation of JNK, ERK, p38, and NF-κB p65 in TRAPS mutant macrophages after TNFα stimulation. TNFα treatment phosphorylated JNK, ERK, p38, and NF-κB p65 in WT macrophages, whereas T79M and G87V mutations reduced the phosphorylation in an allele dose-dependent manner (**Figure 6**). Regarding this aspect, T90I mutant macrophages and WT cells also responded similarly to TNFα (**Figure 6**). The decreased responses to TNFα in TRAPS mutant macrophages were consistent with reduced *Tnf* and *Il1b* mRNA expression, as shown in **Figure 5**.

### Decreased cell surface expression of TNFR1 by TRAPS mutations

**Figure 7:**
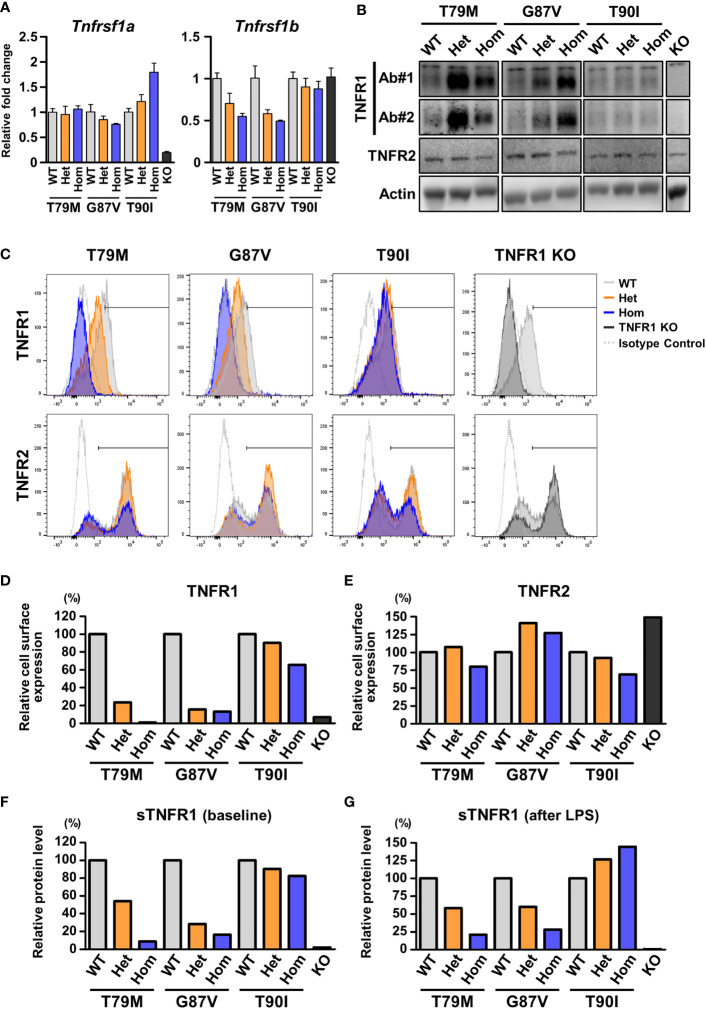
Decreased cell surface expression of TNFR1 in the TRAPS mutant cells. The expression of TNFR1 and TNFR2 were determined in primary murine bone marrow-derived macrophages and peritoneal macrophages. **(A)** mRNA expression levels of *Tnfrsf1a* and *Tnfrsf1b* in bone marrow-derived macrophages. **(B)** Immunoblot analysis of TNFR1 and TNFR2 expression in the bone marrow-derived macrophages. Two different antibodies against TNFR1 were used: Ab#1 (13377, Cell Signaling Technology) recognizes aa 29–43 (extracellular domain), and Ab#2 (AF-425-PB, R&D Systems) recognizes the C-terminal intracellular region. **(C–E)** Flow cytometric analysis of TNFR1 and TNFR2 expression. Peritoneal exudate cells were collected from the indicated mice 3 days after the intraperitoneal administration of thioglycolate. **(C)** Representative histograms of the cell surface expression of TNFR1 and TNFR2. The expression levels of TNFR1 **(D)** and TNFR2 **(E)** on the surface of CD11b-positive cells were determined by flow cytometry. The cells within the horizontal line on each histogram were recognized as TNFR1- or TNFR2-positive cells. The proportions of the positive cells were calculated relative to those of the WT in each strain. The following antibodies were used: TNFR1 (113005, BioLegend) and TNFR2 (113405, BioLegend). Both antibodies recognize the extracellular domains. **(F, G)** ELISA for soluble TNFR1 (sTNFR1) in the culture supernatant of bone marrow-derived macrophages. Culture supernatant was collected before **(F)** and 24 h after **(G)** LPS stimulation. Concentrations of sTNFR1 in the culture supernatant were measured by ELISA, and the levels were calculated relative to that of the WT in each strain. TRAPS, TNF receptor-associated periodic syndrome; LPS, lipopolysaccharide; ELISA, enzyme-linked immunosorbent assay; WT, wild-type; Het, heterozygote; Hom, homozygote.

To clarify the precise mechanisms underlying the decreased response to TNFα in TRAPS mutant macrophages, we evaluated TNFR1 mRNA and protein expression in macrophages. qPCR analysis revealed that the expression levels of *Tnfrsf1a* were not altered in TRAPS mutant macrophages (**Figure 7A**). Next, we analyzed TNFR1 protein levels in the whole-cell lysates of macrophages using two anti-TNFR1 antibodies (Ab), which were made from different immunogens: Ab#1 recognizes aa 29-43 (extracellular domain), and Ab#2 recognizes the C-terminal intracellular region. We found that the expression levels of the TNFR1 protein in the whole-cell lysates were higher in TRAPS mutant macrophages (T79M and G87V) than in WT cells (**Figure 7B**). The T90I mutation did not affect TNFR1 protein levels (**Figure 7B**). T79M and G87V mutations modestly suppressed mRNA expression of *Tnfrsf1b*, which encodes TNFR2 (Fig 7A). TNFR2 protein levels were comparable across WT and *Tnfrsf1a* mutant cells (**Figure 7B**).

Since TRAPS mutations affect the cell surface expression of TNFR1 in overexpression models (2, 4, 6, 27), we assessed the cell surface expression of TNFR1 in peritoneal macrophages using flow cytometry. We found that the cell surface levels of TNFR1 were prominently reduced in TRAPS mutant macrophages compared with those in WT cells in an allele dose-dependent manner (**Figures 7C**, **D**, and **Supplementary Figure 4A**). In contrast, T90I mutation did not significantly affect the cell surface expression of TNFR1 (**Figures 7C**, **D**, and **Supplementary Figure 4A**). Cell surface expression of TNFR2 was not substantially affected by any of the mutations (**Figure 7E** and **Supplementary Figure 4B**).

Next, because sTNFR1 is shed from transmembrane TNFR1 (28), we examined whether sTNFR1 levels are affected by TRAPS mutations. We measured the concentration of sTNFR1 in the culture medium of primary bone marrow-derived macrophages. We found that the concentrations of sTNFR1 were reduced in TRAPS mutant cells compared with those in WT cells at baseline and after LPS stimulation (**Figures 7F**, **G**). The T90I mutation did not affect the concentration of sTNFR1 (**Figures 7F**, **G**). Next, we measured the sTNFR1 concentrations in the sera of TRAPS mutant mice. Serum concentrations of sTNFR1 were reduced in TRAPS mutant mice in an allele dose-dependent manner (**Supplementary Figure 5A**). The reduction in sTNFR1 levels in TRAPS mutant mice was also observed after LPS stimulation (**Supplementary Figure 5B**). These data suggest that decreased cell surface expression of TNFR1 in the TRAPS mutants did not result from increased shedding of membrane-bound TNFR1.

Since TNFR1 protein expression was increased in the whole-cell lysates of TRAPS mutant macrophages (**Figure 7B**), we evaluated the stability of TNFR1 protein in a CHX chase assay. The bone marrow-derived macrophages were treated with CHX, a translation inhibitor, and TNFR1 protein levels were determined by immunoblot analysis. We found that the T79M and G87V mutations delayed TNFR1 degradation (**Supplementary Figures 6A**, **B**) whereas the T90I mutation did not (**Supplementary Figures 6A**, **B**).

Considering the involvement of endoplasmic reticulum (ER) stress has been suggested in the pathogenesis of TRAPS (27), we assessed whether ER stress is increased in TRAPS mutant macrophages. *sXbp1* mRNA expression and ATF-6 and IRE1α were determined as ER stress markers (29). We found that *sXbp1* mRNA expression level or protein levels of ATF-6 and IRE1α were not altered by the TRAPS mutations (**Supplementary Figure 7B**).

### Responsiveness of TRAPS mutant cells to other proinflammatory stimuli

We showed that TRAPS mutations did not enhance LPS-mediated inflammation, but attenuated TNFα-mediated inflammation as presented in **Figures 2**, **5**, **6**. This suggests that LPS and TNFα are probably not essential triggers for the inflammatory attacks seen in patients with TRAPS. Thus, we explored other candidate stimuli that could account for the inflammatory phenotypes in TRAPS.

**Figure 8:**
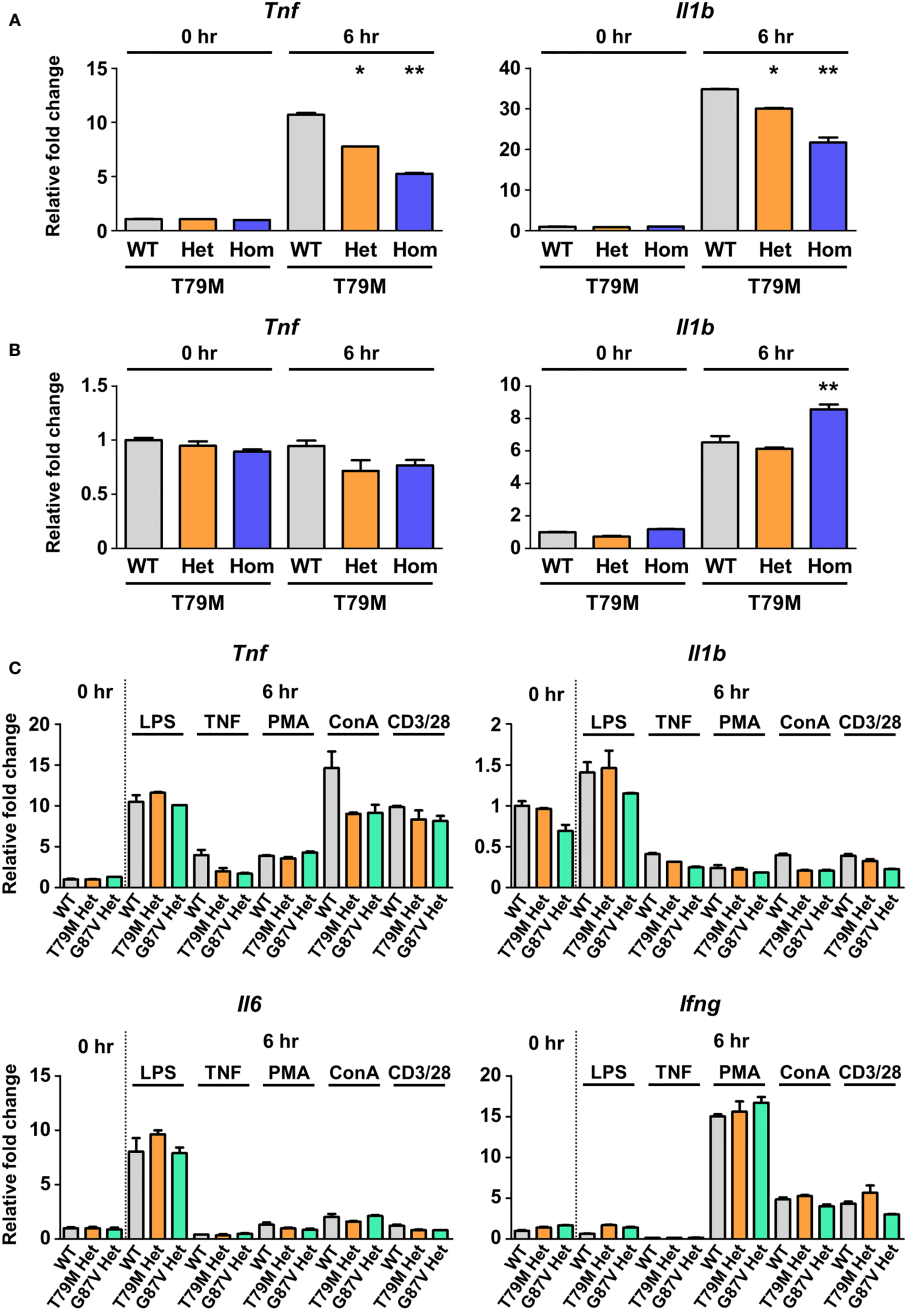
No substantial differences in the responses to inflammatory stimuli in TRAPS mutant macrophages and splenocytes. T79M TRAPS mutant bone marrow-derived macrophages were stimulated with 100 ng/mL LTα **(A)** or 10 mM norepinephrine **(B)**. RNA samples were collected before and 6 h after the stimulation. The mRNA expression levels of *Tnf* and *Il1b* were determined using qPCR. The cell culture experiments were performed separately for each strain. **(C)** Murine splenocytes were collected from indicated mice. The splenocytes were treated with LPS, TNFα, PMA plus ionomycin, ConA, and anti-CD3ϵ/CD28 antibodies. mRNA expression levels of proinflammatory cytokines were determined using qPCR. The WT levels at 0 h were set to 1. *p<0.01 *vs*. T79M WT. **p<0.01 *vs*. T79M WT and Het 6 h after stimulation of LTα or norepinephrine. TRAPS, TNF receptor-associated periodic syndrome; LTα, lymphotoxin α; LPS, lipopolysaccharide; PMA, phorbol 12-myristate 13-acetate; ConA, concanavalin A; qPCR, real-time quantitative polymerase chain reaction; WT, wild-type; Het, heterozygote; Hom, homozygote.

LTα, also known as TNFβ, binds to TNFR1 (30) and induces inflammatory responses in responder cells (31). Therefore, we investigated whether LTα could trigger inflammatory responses in TRAPS mutant cells. Murine bone marrow-derived macrophages obtained from T79M mice were stimulated with LTα. We found that LTα increased *Tnf* and *Il1b* mRNA expression in WT cells, whereas the T79M mutation lowered gene expression in an allele dose-dependent manner (**Figure 8A**). The responses to LTα in TRAPS mutant cells were similar to the responses to TNFα, as shown in **Figure 5**.

One of the other possible triggers for TRAPS is emotional and physical stress (1, 28). The production of norepinephrine is high under stress conditions and is thought to cause proinflammatory responses, such as excessive production of IL-1β, in the microglia (32). We evaluated the responsiveness of TRAPS mutant macrophages to norepinephrine. We found that the mRNA expression of proinflammatory cytokines after norepinephrine stimulation was similar among WT and the TRAPS mutant macrophages (**Figure 8B**).

Next, we used crude populations of murine splenocytes to assess the responsiveness of various immune cells to the inflammatory stimuli. The expression of proinflammatory cytokines was evaluated after primary splenocytes were stimulated with LPS, TNFα, PMA with ionomycin, ConA, and anti-CD3ϵ antibody with anti-CD28 antibody. We found no remarkable differences in expression patterns between WT and TRAPS mutant splenocytes (**Figure 8C**). These findings suggest that the stimuli tested had limited effects on the splenocytes of TRAPS-mutant mice.

## Discussion

In this study, we sought to clarify the pathophysiology of TRAPS using TRAPS mutation knock-in murine models. We found that the TRAPS mutations did not exert excessive inflammatory phenotypes but rather decreased inflammatory responses, demonstrated by reduced lethal responses against LPS and D-galactosamine and diminished TNFα-mediated joint inflammation. In addition, TRAPS-mutant macrophages were resistant to TNFα stimulation. The decreased responses to TNFα in TRAPS mutant macrophages were mediated by reduced cell surface expression of TNFR1.

Our study revealed that T79M and G87V TRAPS mutant mice exhibited reduced inflammatory responses to LPS and D-galactosamine in an allele dose-dependent manner. Simon et al. showed that T79M and C62Y TRAPS mutant homozygotes were completely resistant to the LPS and D-galactosamine stimuli, which is consistent with the results that we obtained using T79M and G87V TRAPS mutant homozygous mice. However, the results of heterozygous mutants in the previous study were different from those in our study. We have shown that TRAPS mutant heterozygotes are tolerant to LPS and D-galactosamine compared with WT mice, whereas Simon et al. showed that heterozygotes were more susceptible to stimulation than WT mice, as demonstrated by increased mortality in heterozygous mice (8). Our data from *in vivo* and *in vitro* analyses suggests that T79M and G87V TRAPS mutant mice are resistant to treatment with LPS and D-galactosamine and also TNFα. However, we cannot exclude the possibility that TRAPS mutant heterozygotes exhibit excessive inflammatory responses to LPS and D-galactosamine under certain conditions, which could predispose the mice to these stimuli. Several differences in experimental settings between our study and the previous study might account for the discrepancy; these include the following: the knock-in method employed to generate mutant mice (genome editing or homologous recombination), nucleotide sequences (no detailed information on the nucleotide sequences to substitute amino acids in the previous study), LPS sources (LPS from *Escherichia coli* O111:B4 or *Escherichia coli* O127:B8), housing conditions (including environmental pathogens and diet), age of the mice, and observation period (no detailed description in the previous study). As we did not identify the exact candidate stimulant for TRAPS, further studies are needed.

T79M and G87V TRAPS mutations decreased the cell surface expression of TNFR1 in macrophages. This finding is consistent with previous studies on TRAPS-mutant TNFR1 overexpressing cells (4, 5, 27). We also found that sTNFR1 levels were not increased in the culture supernatant of mutant macrophages or in the sera of mutant mice. These data suggest that the decreased membrane-bound TNFR1 was not due to an increase in the shedding of membrane-bound TNFR1. Furthermore, we found that the T79M and G87V mutations delayed the degradation of TNFR1, resulting in the increased stability of mutant TNFR1. These findings suggest the TNFR1 protein accumulated in the cytoplasm of the cells. Indeed, intracellular accumulation has been reported in mutant TNFR1 overexpressing cell models (5, 27). Furthermore, previous studies have shown aberrant accumulation of TNFR1 in the ER, and it has been proposed that ER stress induces MAPK activation at baseline and predisposes the cells to inflammation (5, 33). However, we did not observe baseline activation of MAPKs in the mutant cells (**Figure 4**, **6**) or elevation of ER stress markers (**Supplementary Figure 7**). Although we found decreased cell surface expression of TNFR1 and increased TNFR1 stability in TRAPS mutant macrophages, their pathological implication in TRAPS-associated inflammation requires validation in future studies.

TNFα binds to both TNFR1 and TNFR2 (30), and both exert proinflammatory activity (34). As shown in this study, TNFR1 is less likely to mediate inflammatory responses by binding to TNFα. We hypothesized that TNFR2 could induce inflammatory reactions in TRAPS mutant cells. Because cell surface expression of TNFR1 is prominently suppressed in TRAPS mutant homozygotes, we speculated that TNFR2-mediated responses are alternatively activated in homozygotes and expected that inflammatory responses would be induced in response to TNFα *via* TNFR2. Contrary to our expectation, TNFα did not induce inflammatory responses in TRAPS homozygotes; rather, the responses were strikingly suppressed to a similar extent as in TNFR1 KO cells. Although we tested LTα, another TNFR1/TNFR2 agonist, the responsiveness of TRAPS mutant cells to LTα was similar to that of TNFα. These findings suggest that TNFR2-mediated pathways are not substantially affected by TRAPS mutations.

In clinical practice, inflammatory attacks in patients with TRAPS are supposedly precipitated by minor infections, trauma, hormonal changes, emotional and physical stress, fatigue, and vaccinations (28). However, the exact triggering factors remain unknown in most cases. In this study, we tested LPS, D-galactosamine, TNFα, ATP, LTα, norepinephrine, PMA, ConA, and anti-CD3/28 antibodies; however, they did not induce excessive inflammatory responses in TRAPS mutants beyond those observed in WT mice and cells. Other than the stimuli tested in this study, other proinflammatory factors may exert inflammatory responses during TRAPS, for example, TLR ligands other than LPS, inflammasome inducers other than ATP, and inflammatory cytokines, such as interferon-γ (IFNγ) (4, 35). Moreover, not a single stimulation, but a combination of stimuli under a stressful environment, might trigger inflammatory attacks. Further studies are needed to identify uncharacterized inflammatory factors.

In this study, we analyzed the phenotypes of G87V and T90I mutant mice for the first time. The G87V mutation was identified by our group in two TRAPS patients (4), whereas the T90I mutation was considered a low-penetrance mutation and is currently recognized as a variant of unknown significance (2). We found that G87V mutant mice and macrophages behaved similarly to previously identified pathogenic T79M mutant mice and macrophages. These findings are in accordance with our previous results, confirmed in mutant TNFR1 overexpression models (4), supporting the pathogenic impact of the G87V mutation in TRAPS. In contrast, T90I mutant mice and macrophages did not exhibit the same dynamics as T79M or G87V mutant mice and macrophages; instead, T90I mutants behaved similarly to the WT mice and cells. These results suggest that the T90I mutation is unlikely to be responsible for TRAPS, although we cannot rule out the involvement of T90I mutation-specific inflammatory mechanisms other than those evaluated in this study.

For the assessment of the validity of our results, we believe that evaluation in human specimens would be necessary. Previously, we analyzed the functions of peripheral blood mononuclear cells (PBMCs) from TRAPS patients harboring a G87V heterozygous mutation (4). In the study, we tested the effect of TNFα on the secretion of inflammatory cytokines from TRAPS PBMCs and found that TNFα did not enhance the secretion of inflammatory cytokines compared with that in healthy control PBMCs (4). Our previous finding that TNFα is unlikely to initiate TRAPS-associated inflammation is consistent with our present findings based on murine models; however, the responsiveness to TNFα in the TRAPS PBMC cultures was not decreased as observed in TRAPS heterozygous mutant mice in our current study. These findings suggest that responsiveness to stimuli, including TNFα, is different between humans and mice, possibly due to different genetic backgrounds. Therefore, further studies are needed using human samples and murine models to clarify the pathogenesis of TRAPS by integrating findings from human and murine models.

In conclusion, TRAPS mutations did not augment responsiveness to TNFα, LPS, or other stimuli. This suggests that inflammation in TRAPS can be induced by unconfirmed disease-specific proinflammatory factors other than these stimuli. Further studies are required to elucidate the mechanisms underlying TRAPS.

## Data availability statement

The raw data supporting the conclusions of this article will be made available by the authors, without undue reservation.

## Ethics statement

This study was reviewed and approved by the Institutional Animal Care and Use Committee of Kawasaki Medical School.

## Author contributions

Conceptualization: TA, YM, and TM. Methodology: TA, MM, and TM. Resources: TWM and KIs. Acquisition and analysis of data: TA, ST, and TM. Interpretation of data: TA, SH-A, KIk, HH, ST, AY, MI, MM, TWM, KN, KIs, YM, TM. Writing-original draft preparation: TA and TM. Writing-review and editing: TA, SH-A, KIk, HH, ST, AY, MI, MM, TWM, KN, KIs, YM, and TM. Funding Acquisition: TA, SH-A, YM, and TM. All authors have contributed to the manuscript and approved the submitted version.

## Funding

This work was supported by JSPS Grant-in-Aid for Scientific Research [19K08923 to HH, 21K08484 to TM, 20K08814 to YM, and 20K17442 to SH-A], Health Labour Sciences Research Grant [20FC1047 to TM], Mishima Kaiun Memorial Foundation [research grant to TA], Kawasaki Medical School [R03G-004 to TA], Eli Lilly Japan [Eli Lilly Japan KK Research Grant 2021 to TM], and UCB Japan [research grant to TM].

## Conflict of interest

TA, SH-A, KN, and YM received scholarship donations from AbbVie, Asahi Kasei, Ayumi and Chugai.

The remaining authors declare that the research was conducted in the absence of any commercial or financial relationships that could be construed as a potential conflict of interest.

## Publisher’s note

All claims expressed in this article are solely those of the authors and do not necessarily represent those of their affiliated organizations, or those of the publisher, the editors and the reviewers. Any product that may be evaluated in this article, or claim that may be made by its manufacturer, is not guaranteed or endorsed by the publisher.

## Supplementary material

Supplementary Figure 1
Generation of TNFRSF1A mutant mice by CRISPR/Cas9 system. **(A)** Structure of Tnfrsf1a. Nucleotide sequences of exon 3 and amino acid sequence alignments are shown. TM, transmembrane domain. **(B)** Designs of guide RNA and single-stranded oligodeoxynucleotides for knocking in the indicated mutations. Nucleotide mutations after targeting are presented in red. PAM, protospacer-adjacent motif. **(C)** RFLP analysis of Tnfrsf1a T79M, G87V, and T90I mutant mice. DNA samples were digested using CviAII, BstPI, and AluI for the corresponding mice. **(D)** Sanger sequencing chromatograms of homozygous T79M, G87V, and T90I mutant mice. RFLP, restriction fragment length polymorphism; CRISPR, clustered regularly interspaced short palindromic repeats; WT, wild type; Het, heterozygote; Hom, homozygote.

Supplementary Figure 2
The Tlr4 mRNA expression in primary murine bone marrow-derived macrophages. Bone marrow-derived macrophages were cultured with M-CSF in the absence of additional stimuli. Tlr4 mRNA expression was determined by qPCR. The level for each WT was set at 1. Values are presented as mean ± standard deviation. WT, wild type; Het, heterozygote; Hom, homozygote; KO, knockout; TNFR1, tumor necrosis factor (TNF) receptor type I; M-CSF, macrophage colony-stimulating factor; qPCR, real-time quantitative polymerase chain reaction.

Supplementary Figure 3
The concentrations of IL-1β in the culture supernatant of T79M mutant primary bone marrow-derived macrophages. T79M mutant murine macrophages were stimulated with **(A)** TNFα (100 ng/mL), **(B)** LPS (100 ng/mL), and **(C)** LPS with ATP (5 mM). The culture supernatant was collected at the indicated time points. The concentration of IL-1β in the culture supernatant was measured using ELISA. IL, interleukin, ELISA, enzyme-linked immunosorbent assay; LPS, lipopolysaccharide; ATP, adenosine triphosphate; WT, wild-type; Het, heterozygote; Hom, homozygote.

Supplementary Figure 4
Flow cytometry analysis of TNFR1 and TNFR2 in peritoneal macrophages. Flow cytometric analysis of TNFR1 and TNFR2 expression. Peritoneal exudate cells were collected from the indicated mice 3 days after the intraperitoneal administration of thioglycolate. The expression levels of TNFR1 and TNFR2 on the surface of CD11b-positive cells were determined by flow cytometry. **(A)** MFI of TNFR1. The MFI of the whole cells on the histogram (Figure 7C) was measured. **(B)** MFI of TNFR2. The MFI of the indicated cells gated on Figure 7C was measured. TNFR1, tumor necrosis factor (TNF) receptor type I; TNFR2, TNF receptor type II; MFI, mean fluorescence intensity; WT, wild type; Het, heterozygote; Hom, homozygote.

Supplementary Figure 5
The serum concentrations of sTNFR1 in the TRAPS mutant mice. (A) Serum samples were collected from T79M and G87V TRAPS mutant mice at 18 weeks. The serum concentrations of sTNFR1 were measured using ELISA. **(B)** Serum samples were collected from T79M mutant mice at the age of 18 weeks 2 hours later the intraperitoneal administration with LPS (2 μg/mouse). Each dot represents an individual mouse. Bars indicate the mean values. ELISA, enzyme-linked immunosorbent assay; LPS, lipopolysaccharide; WT, wild type; Het, heterozygote; Hom, homozygote.

Supplementary Figure 6
Increased stability of TNFR1 protein in TRAPS mutant primary bone marrow-derived macrophages. Primary bone marrow-derived macrophages were treated with 20 μg/mL cycloheximide, and protein samples were collected using RIPA buffer at the indicated time points. **(A)** Immunoblot analysis of the TNFR1 protein. The TNFR1 protein was detected using an anti-TNFR1 antibody (13377, Cell Signaling Technology). **(B)** TNFR1 protein levels were quantified using Image Studio Lite (Ver 5.2, LI-COR). The level at 0 h for each genotype was used as a standard for quantification. TNFR1, tumor necrosis factor (TNF) receptor type I; TRAPS, TNF receptor-associated periodic syndrome; WT, wild-type; Het, heterozygote; Hom, homozygote.

Supplementary Figure 7
ER stress markers in TRAPS mutant primary bone marrow-derived macrophages. Primary bone marrow-derived macrophages were cultured with M-CSF in the absence of additional stimuli. **(A)** Unspliced Xbp1 (uXbp1) and spliced Xbp1 (sXbp1) mRNA expression levels were determined by qPCR. The level for each WT was set at 1. Values are presented as mean ± standard deviation. **(B)** ATF-6 and IRE1α protein expression levels were detected by Western blot. ER, endoplasmic reticulum; WT, wild-type; Het, heterozygote; Hom, homozygote.

## References

[1] Cudrici C Deuitch N Aksentijevich I. Revisiting TNF receptor-associated periodic syndrome (TRAPS): Current perspectives. Int J Mol Sci (2020) 21(9):3263. doi: 10.3390/ijms21093263 PMC7246474 32380704

[2] Ueda N Ida H Washio M Miyahara H Tokunaga S Tanaka F. Clinical and genetic features of patients with Tnfrsf1a variants in Japan: Findings of a nationwide survey. Arthritis Rheumatol (2016) 68(11):2760–71. doi: 10.1002/art.39793 27332769

[3] Sarrauste de Menthière C Terrière S Pugnère D Ruiz M Demaille J Touitou I. Infevers: The registry for fmf and hereditary inflammatory disorders mutations. Nucleic Acids Res (2003) 31(1):282–5. doi: 10.1093/nar/gkg031 PMC165478 12520003

[4] Tsuji S Matsuzaki H Iseki M Nagasu A Hirano H Ishihara K. Functional analysis of a novel G87V TNFRSF1A mutation in patients with TNF receptor-associated periodic syndrome. Clin Exp Immunol (2019) 198(3):416–29. doi: 10.1111/cei.13365 PMC6857086 31429073

[5] Lobito AA Kimberley FC Muppidi JR Komarow H Jackson AJ Hull KM. Abnormal disulfide-linked oligomerization results in er retention and altered signaling by TNFR1 mutants in TNFR1-associated periodic fever syndrome (TRAPS). Blood (2006) 108(4):1320–7. doi: 10.1182/blood-2005-11-006783 PMC1895878 16684962

[6] Bachetti T Chiesa S Castagnola P Bani D Di Zanni E Omenetti A. Autophagy contributes to inflammation in patients with TNFR-associated periodic syndrome (TRAPS). Ann Rheum Dis (2013) 72(6):1044–52. doi: 10.1136/annrheumdis-2012-201952 23117241

[7] Yousaf N Gould DJ Aganna E Hammond L Mirakian RM Turner MD. Tumor necrosis factor receptor I from patients with tumor necrosis factor receptor-associated periodic syndrome interacts with wild-type tumor necrosis factor receptor I and induces ligand-independent NF-κβ activation. Arthritis Rheum (2005) 52(9):2906–16. doi: 10.1002/art.21268 16142754

[8] Simon A Park H Maddipati R Lobito AA Bulua AC Jackson AJ. Concerted action of wild-type and mutant TNF receptors enhances inflammation in TNF receptor 1-associated periodic fever syndrome. Proc Natl Acad Sci U S A (2010) 107(21):9801–6. doi: 10.1073/pnas.0914118107 PMC2906866 20457915

[9] McDermott MF Aksentijevich I Galon J McDermott EM Ogunkolade BW Centola M. Germline mutations in the extracellular domains of the 55 kda TNF receptor, TNFR1, define a family of dominantly inherited autoinflammatory syndromes. Cell (1999) 97(1):133–44. doi: 10.1016/s0092-8674(00)80721-7 10199409

[10] Hashimoto M Takemoto T. Electroporation enables the efficient mRNA delivery into the mouse zygotes and facilitates CRISPR/Cas9-based genome editing. Sci Rep (2015) 5:11315. doi: 10.1038/srep11315 26066060 PMC4463957

[11] Hashimoto M Yamashita Y Takemoto T. Electroporation of Cas9 protein/SgRNA into early pronuclear zygotes generates non-mosaic mutants in the mouse. Dev Biol (2016) 418(1):1–9. doi: 10.1016/j.ydbio.2016.07.017 27474397

[12] Pfeffer K Matsuyama T Kündig TM Wakeham A Kishihara K Shahinian A. Mice deficient for the 55 kd tumor necrosis factor receptor are resistant to endotoxic shock, yet succumb to l. monocytogenes infection. Cell (1993) 73(3):457–67. doi: 10.1016/0092-8674(93)90134-c 8387893

[13] Rothe J Lesslauer W Lötscher H Lang Y Koebel P Köntgen F. Mice lacking the tumour necrosis factor receptor 1 are resistant to TNF-mediated toxicity but highly susceptible to infection by listeria monocytogenes. Nature (1993) 364(6440):798–802. doi: 10.1038/364798a0 8395024

[14] Hayward MD Jones BK Saparov A Hain HS Trillat AC Bunzel MM. An extensive phenotypic characterization of the hTNFα transgenic mice. BMC Physiol (2007) 7:13. doi: 10.1186/1472-6793-7-13 18070349 PMC2222242

[15] Mukai T Gallant R Ishida S Kittaka M Yoshitaka T Fox DA. Loss of SH3 domain-binding protein 2 function suppresses bone destruction in tumor necrosis factor-driven and collagen-induced arthritis in mice. Arthritis Rheumatol (2015) 67(3):656–67. doi: 10.1002/art.38975 PMC4342302 25470448

[16] Akagi T Mukai T Mito T Kawahara K Tsuji S Fujita S. Effect of angiotensin II on bone erosion and systemic bone loss in mice with tumor necrosis factor-mediated arthritis. Int J Mol Sci (2020) 21(11):4145. doi: 10.3390/ijms21114145 PMC7312645 32532031

[17] Mukai T Akagi T Hiramatsu Asano S Tosa I Ono M Kittaka M. Imatinib has minimal effects on inflammatory and osteopenic phenotypes in a murine cherubism model. Oral Dis (2021). doi: 10.1111/odi.14073 PMC9076755 34743383

[18] Fujita S Mukai T Mito T Kodama S Nagasu A Kittaka M. Pharmacological inhibition of tankyrase induces bone loss in mice by increasing osteoclastogenesis. Bone (2018) 106:156–66. doi: 10.1016/j.bone.2017.10.017 PMC6912859 29055830

[19] Mukai T Ishida S Ishikawa R Yoshitaka T Kittaka M Gallant R. SH3BP2 cherubism mutation potentiates TNF-α-induced osteoclastogenesis *via* NFATc1 and TNFα-mediated inflammatory bone loss. J Bone Miner Res (2014) 29(12):2618–35. doi: 10.1002/jbmr.2295 PMC4262741 24916406

[20] Krawiec JA Chen H Alom-Ruiz S Jaye M. Modified paxgene method allows for isolation of high-integrity total RNA from microlitre volumes of mouse whole blood. Lab Anim (2009) 43(4):394–8. doi: 10.1258/la.2008.0070157 19502296

[21] Kawahara K Mukai T Iseki M Nagasu A Nagasu H Akagi T. SH3BP2 deficiency ameliorates murine systemic lupus erythematosus. Int J Mol Sci (2021) 22(8):4169. doi: 10.3390/ijms22084169 33920631 PMC8073120

[22] Ikeda K Morizane S Akagi T Hiramatsu-Asano S Tachibana K Yahagi A. Obesity and dyslipidemia synergistically exacerbate psoriatic skin inflammation. Int J Mol Sci (2022) 23(8):4312. doi: 10.3390/ijms23084312 35457132 PMC9032572

[23] Dong W Song E Song Y. Co-Administration of lipopolysaccharide and d-galactosamine induces genotoxicity in mouse liver. Sci Rep (2021) 11(1):1733. doi: 10.1038/s41598-021-81383-5 33462304 PMC7814041

[24] Ishizaki-Koizumi S Sonaka I Takei Y Ikejima K Sato N. The glycine analogue, aminomethanesulfonic acid, inhibits LPS-induced production of TNFα in isolated rat kupffer cells and exerts hepatoprotective effects in mice. Biochem Biophys Res Commun (2004) 322(2):514–9. doi: 10.1016/j.bbrc.2004.07.147 15325260

[25] Netea MG Nold-Petry CA Nold MF Joosten LA Opitz B van der Meer JH. Differential requirement for the activation of the inflammasome for processing and release of IL-1β in monocytes and macrophages. Blood (2009) 113(10):2324–35. doi: 10.1182/blood-2008-03-146720 PMC2652374 19104081

[26] Greco E Aita A Galozzi P Gava A Sfriso P Negm OH. The novel S59P mutation in the TNFRSF1A gene identified in an adult onset TNF receptor associated periodic syndrome (TRAPS) constitutively activates NF-κβ pathway. Arthritis Res Ther (2015) 17(1):93. doi: 10.1186/s13075-015-0604-7 25888769 PMC4416318

[27] Dickie LJ Aziz AM Savic S Lucherini OM Cantarini L Geiler J. Involvement of X-box binding protein 1 and reactive oxygen species pathways in the pathogenesis of tumour necrosis factor receptor-associated periodic syndrome. Ann Rheum Dis (2012) 71(12):2035–43. doi: 10.1136/annrheumdis-2011-201197 22679299

[28] Hull KM Drewe E Aksentijevich I Singh HK Wong K McDermott EM. The TNF receptor-associated periodic syndrome (TRAPS): Emerging concepts of an autoinflammatory disorder. Medicine (Baltimore) (2002) 81(5):349–68. doi: 10.1097/00005792-200209000-00002 12352631

[29] Korennykh A Walter P. Structural basis of the unfolded protein response. Annu Rev Cell Dev Biol (2012) 28:251–77. doi: 10.1146/annurev-cellbio-101011-155826 23057742

[30] Remouchamps C Boutaffala L Ganeff C Dejardin E. Biology and signal transduction pathways of the lymphotoxin-Aβ/Ltβr system. Cytokine Growth Factor Rev (2011) 22(5-6):301–10. doi: 10.1016/j.cytogfr.2011.11.007 22152226

[31] Hirose T Fukuma Y Takeshita A Nishida K. The role of lymphotoxin-A in rheumatoid arthritis. Inflamm Res (2018) 67(6):495–501. doi: 10.1007/s00011-018-1139-6 29541795

[32] Tomozawa Y Yabuuchi K Inoue T Satoh M. Participation of cAMP and cAMP-dependent protein kinase in beta-adrenoceptor-mediated interleukin-1 beta mRNA induction in cultured microglia. Neurosci Res (1995) 22(4):399–409. doi: 10.1016/0168-0102(95)00922-g 7478305

[33] Bulua AC Simon A Maddipati R Pelletier M Park H Kim KY. Mitochondrial reactive oxygen species promote production of proinflammatory cytokines and are elevated in TNFR1-associated periodic syndrome (TRAPS). J Exp Med (2011) 208(3):519–33. doi: 10.1084/jem.20102049 PMC3058571 21282379

[34] Medler J Wajant H. Tumor necrosis factor receptor-2 (Tnfr2): An overview of an emerging drug target. Expert Opin Ther Targets (2019) 23(4):295–307. doi: 10.1080/14728222.2019.1586886 30856027

[35] Negm OH Singh S Abduljabbar W Hamed MR Radford P McDermott EM. Patients with tumour necrosis factor (Tnf) receptor-associated periodic syndrome (TRAPS) are hypersensitive to toll-like receptor 9 stimulation. Clin Exp Immunol (2019) 197(3):352–60. doi: 10.1111/cei.13306 PMC6693964 31009059

