# Supplementary figures and images for: TRAPS mutations in Tnfrsf1a decrease the responsiveness to TNFα via reduced cell surface expression of TNFR1
Source: Front Immunol. 2022 Jul 22;13:926175. doi: 10.3389/fimmu.2022.926175 (PMC9355097; doi:10.3389/fimmu.2022.926175)

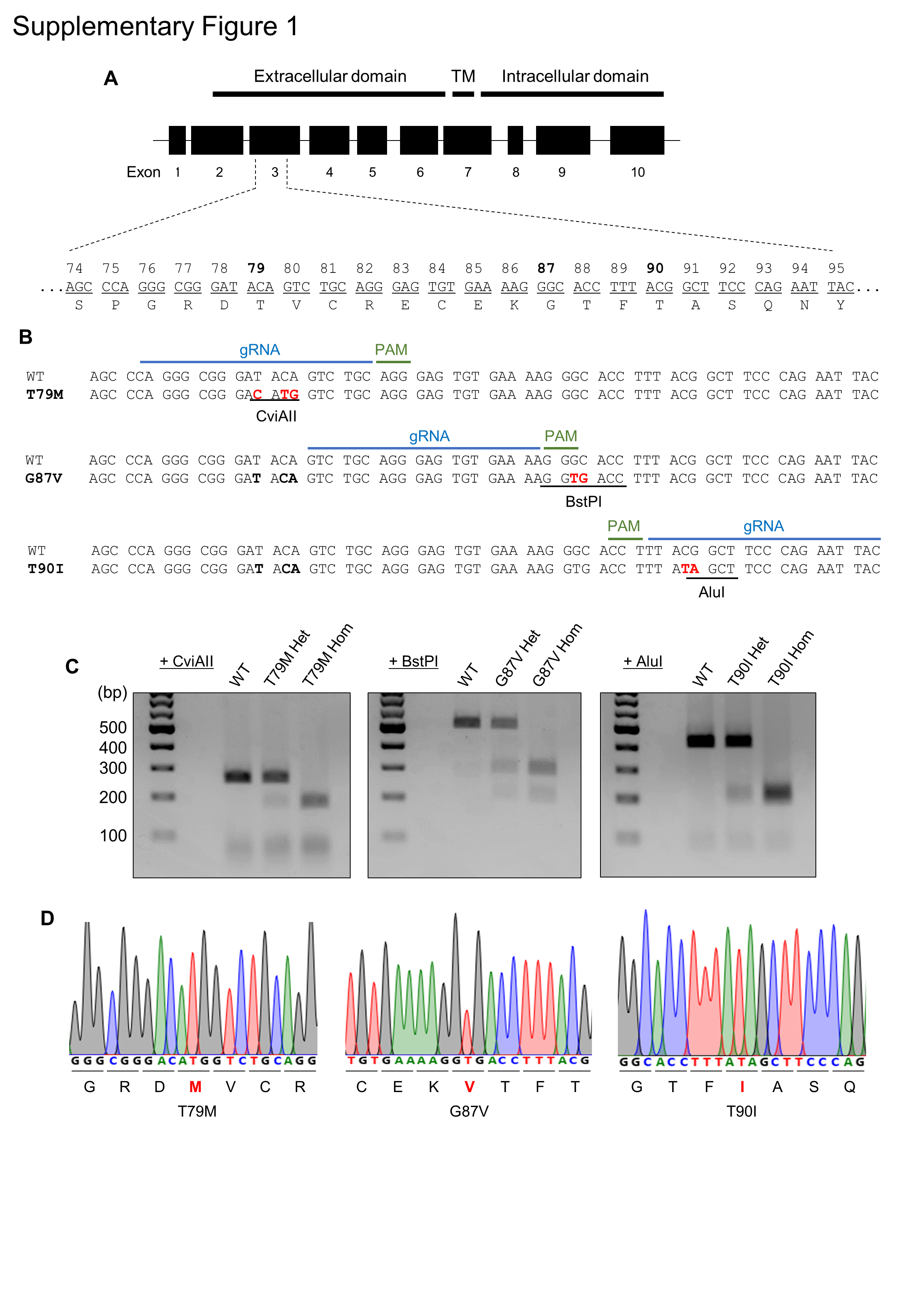

Supplement: Supplementary Figure 1 — Generation of TNFRSF1A mutant mice by CRISPR/Cas9 system. (A) Structure of Tnfrsf1a. Nucleotide sequences of exon 3 and amino acid sequence alignments are shown. TM, transmembrane domain. (B) Designs of guide RNA and single-stranded oligodeoxynucleotides for knocking in the indicated mutations. Nucleotide mutations after targeting are presented in red. PAM, protospacer-adjacent motif. (C) RFLP analysis of Tnfrsf1a T79M, G87V, and T90I mutant mice. DNA samples were digested using CviAII, BstPI, and AluI for the corresponding mice. (D) Sanger sequencing chromatograms of homozygous T79M, G87V, and T90I mutant mice. RFLP, restriction fragment length polymorphism; CRISPR, clustered regularly interspaced short palindromic repeats; WT, wild type; Het, heterozygote; Hom, homozygote. [file Image_1.tif]

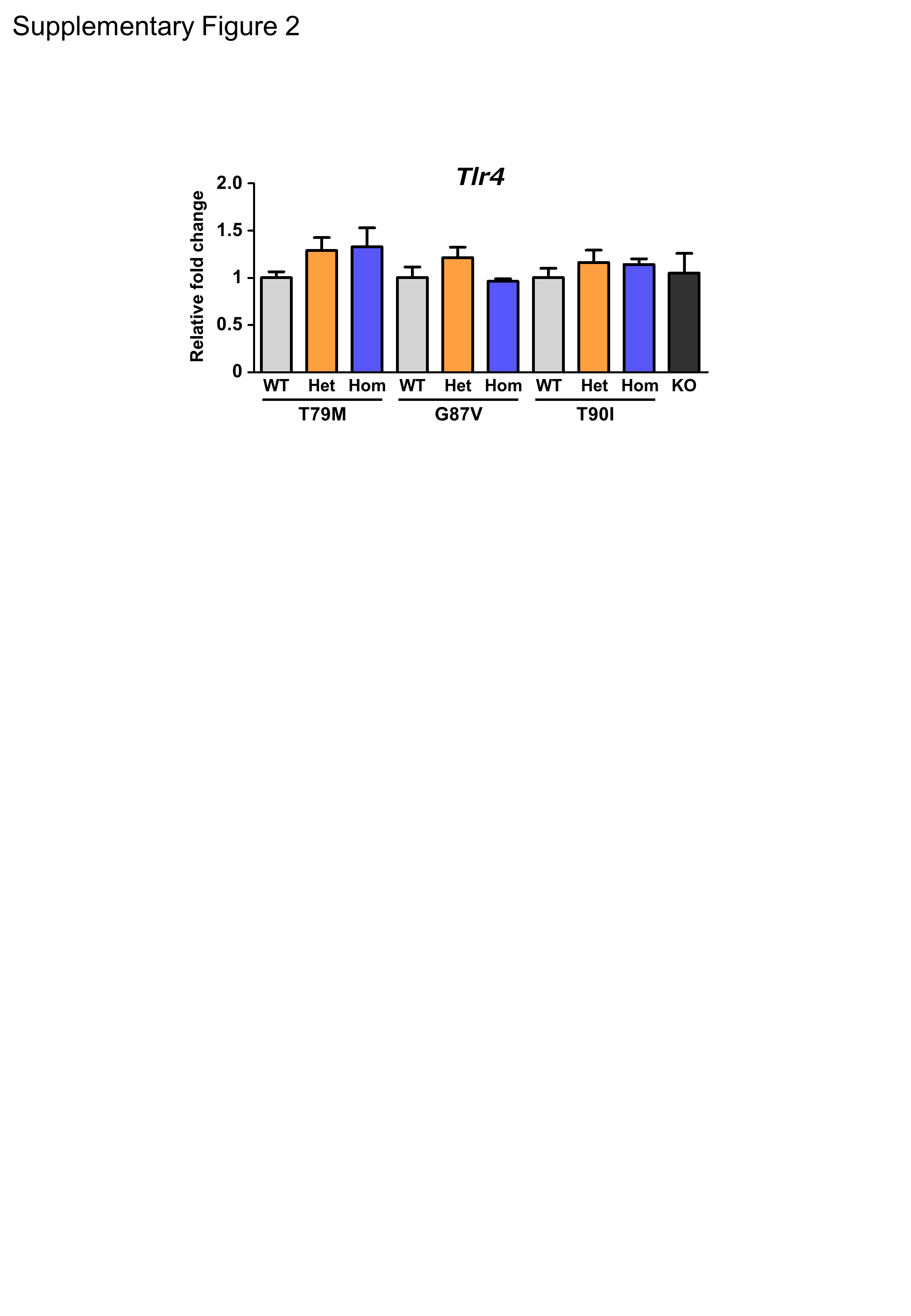

Supplement: Supplementary Figure 2 — The Tlr4 mRNA expression in primary murine bone marrow-derived macrophages. Bone marrow-derived macrophages were cultured with M-CSF in the absence of additional stimuli. Tlr4 mRNA expression was determined by qPCR. The level for each WT was set at 1. Values are presented as mean ± standard deviation. WT, wild type; Het, heterozygote; Hom, homozygote; KO, knockout; TNFR1, tumor necrosis factor (TNF) receptor type I; M-CSF, macrophage colony-stimulating factor; qPCR, real-time quantitative polymerase chain reaction. [file Image_2.tif]

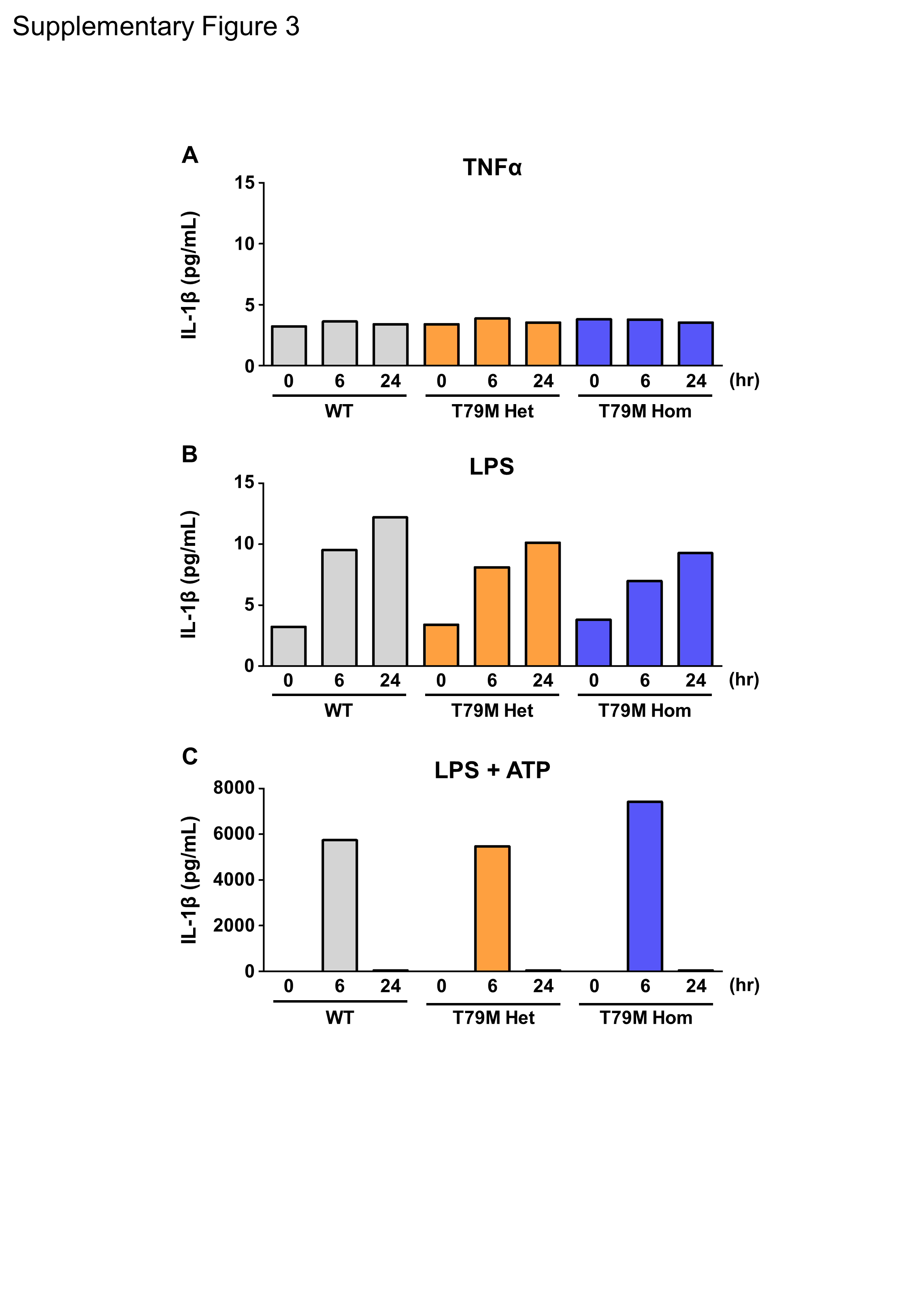

Supplement: Supplementary Figure 3 — The concentrations of IL-1β in the culture supernatant of T79M mutant primary bone marrow-derived macrophages. T79M mutant murine macrophages were stimulated with (A) TNFα (100 ng/mL), (B) LPS (100 ng/mL), and (C) LPS with ATP (5 mM). The culture supernatant was collected at the indicated time points. The concentration of IL-1β in the culture supernatant was measured using ELISA. IL, interleukin, ELISA, enzyme-linked immunosorbent assay; LPS, lipopolysaccharide; ATP, adenosine triphosphate; WT, wild-type; Het, heterozygote; Hom, homozygote. [file Image_3.tif]

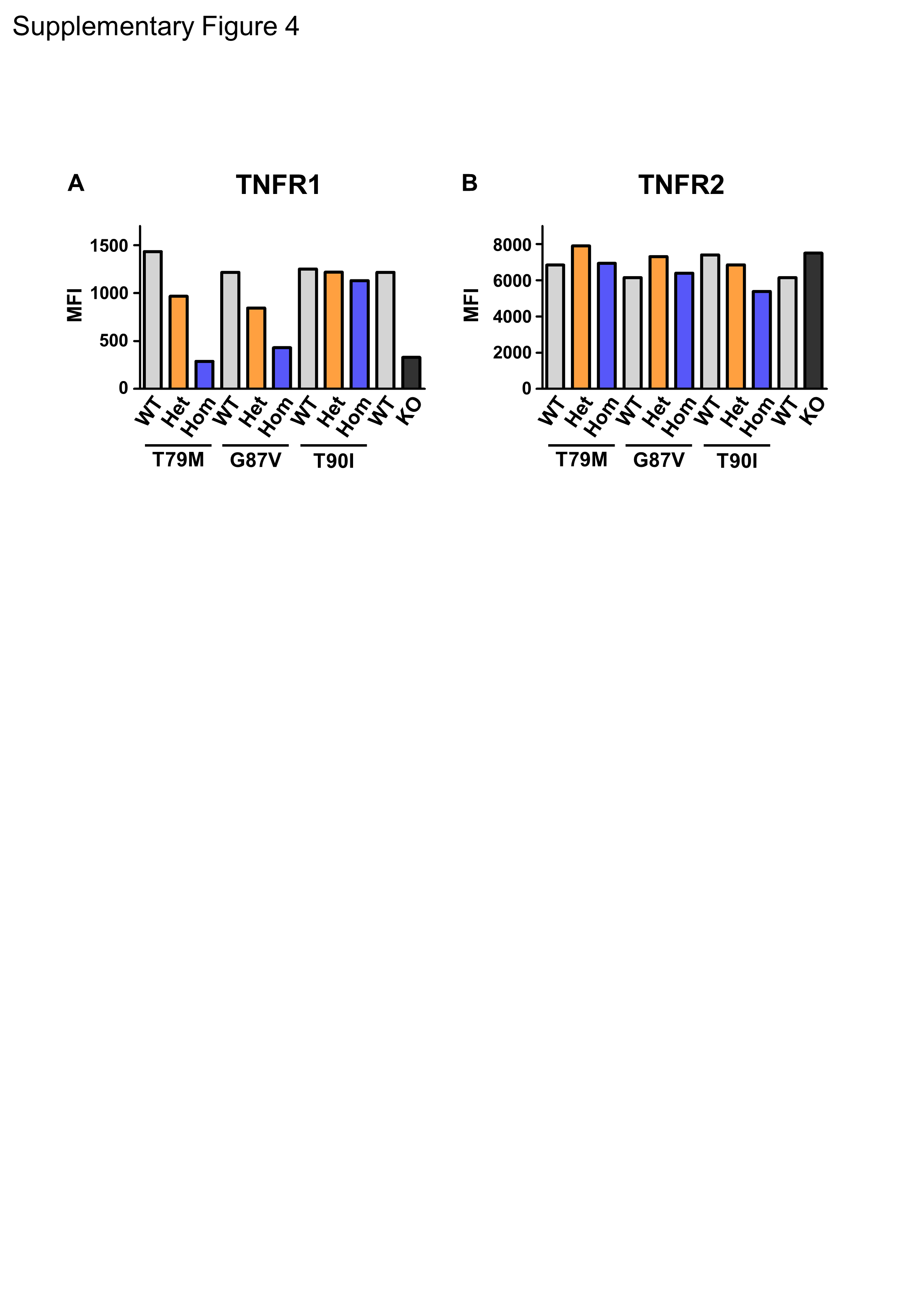

Supplement: Supplementary Figure 4 — Flow cytometry analysis of TNFR1 and TNFR2 in peritoneal macrophages. Flow cytometric analysis of TNFR1 and TNFR2 expression. Peritoneal exudate cells were collected from the indicated mice 3 days after the intraperitoneal administration of thioglycolate. The expression levels of TNFR1 and TNFR2 on the surface of CD11b-positive cells were determined by flow cytometry. (A) MFI of TNFR1. The MFI of the whole cells on the histogram (Figure 7C) was measured. (B) MFI of TNFR2. The MFI of the indicated cells gated on Figure 7C was measured. TNFR1, tumor necrosis factor (TNF) receptor type I; TNFR2, TNF receptor type II; MFI, mean fluorescence intensity; WT, wild type; Het, heterozygote; Hom, homozygote. [file Image_4.tif]

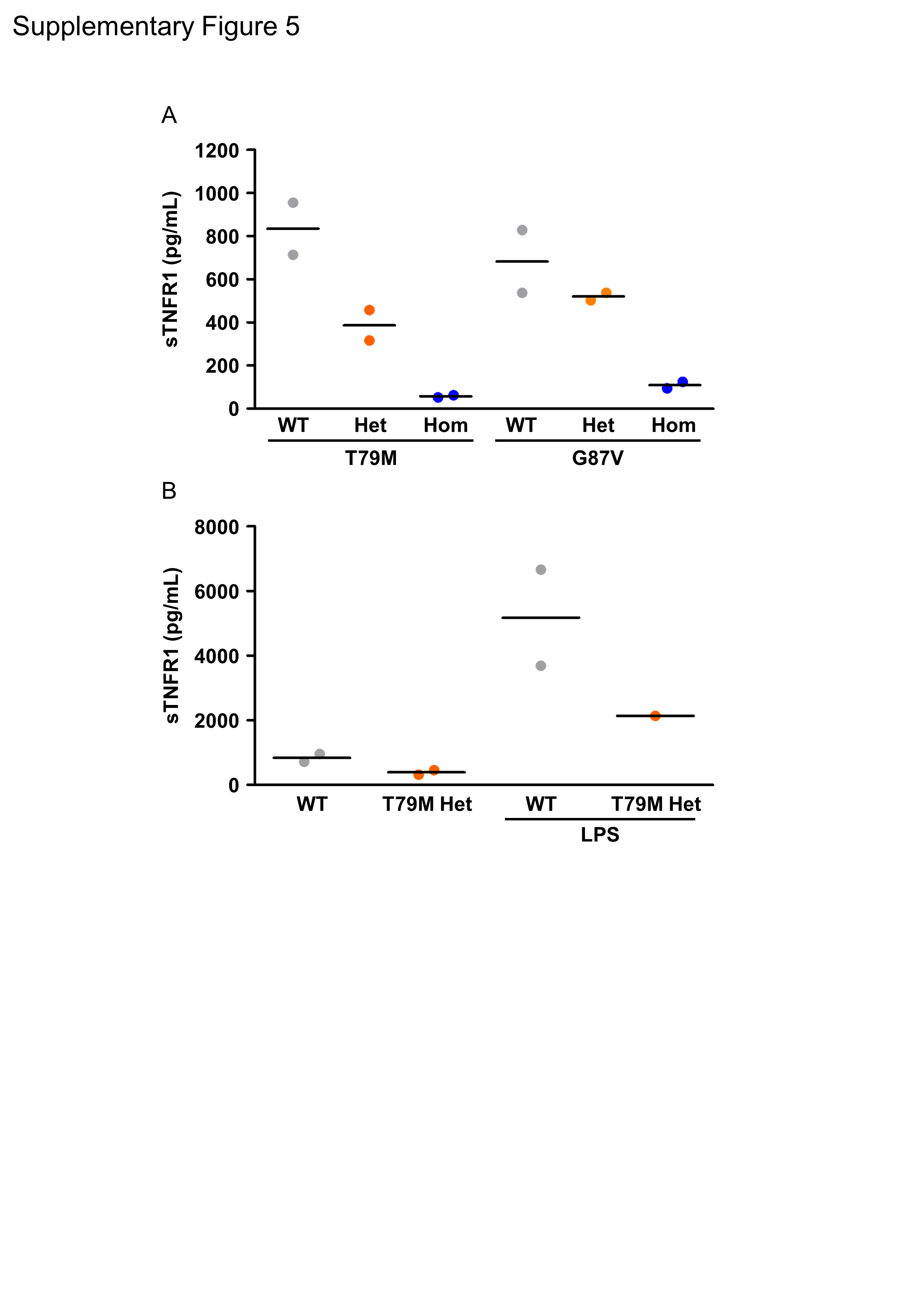

Supplement: Supplementary Figure 5 — The serum concentrations of sTNFR1 in the TRAPS mutant mice. (A) Serum samples were collected from T79M and G87V TRAPS mutant mice at 18 weeks. The serum concentrations of sTNFR1 were measured using ELISA. (B) Serum samples were collected from T79M mutant mice at the age of 18 weeks 2 hours later the intraperitoneal administration with LPS (2 μg/mouse). Each dot represents an individual mouse. Bars indicate the mean values. ELISA, enzyme-linked immunosorbent assay; LPS, lipopolysaccharide; WT, wild type; Het, heterozygote; Hom, homozygote. [file Image_5.tif]

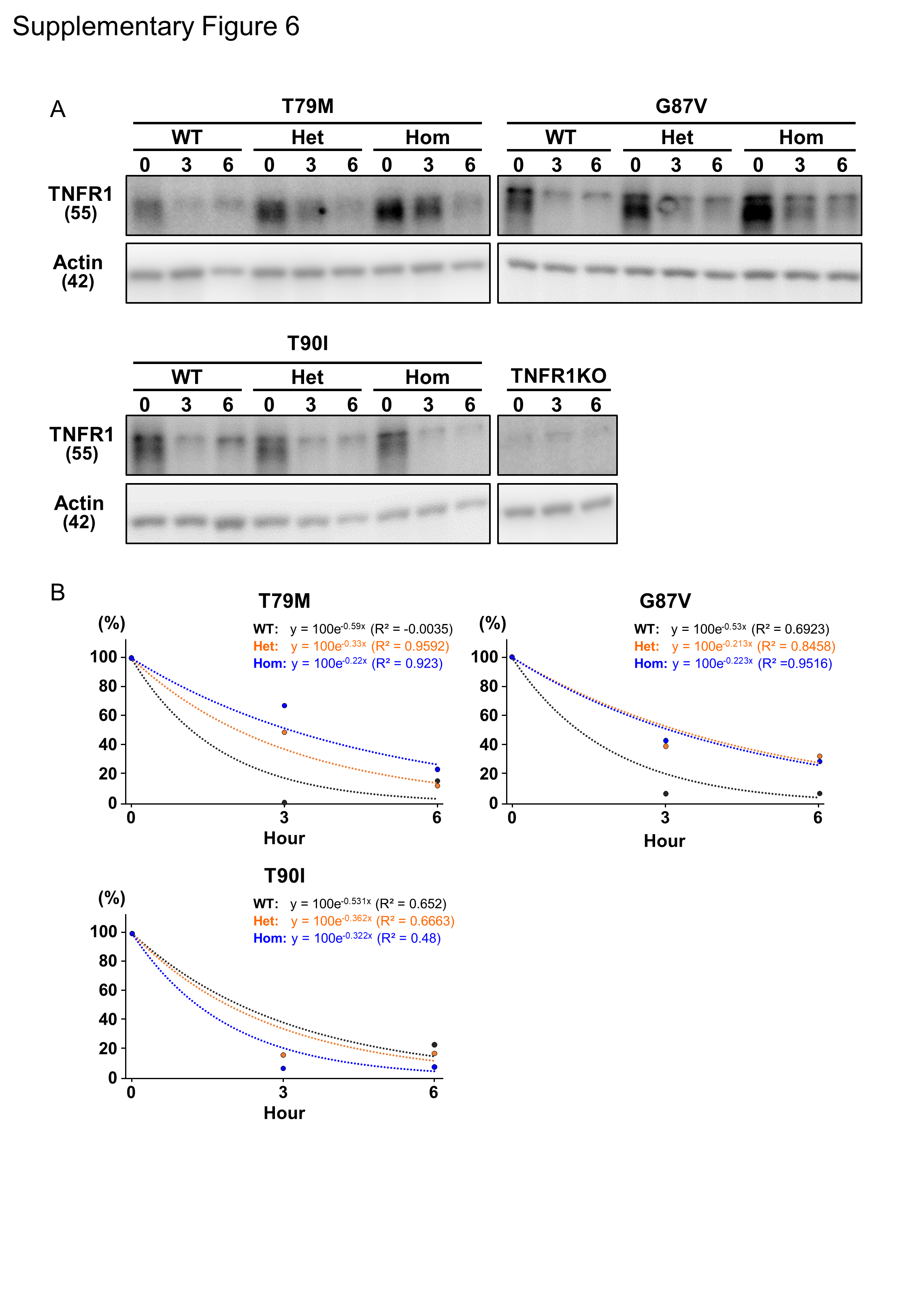

Supplement: Supplementary Figure 6 — Increased stability of TNFR1 protein in TRAPS mutant primary bone marrow-derived macrophages. Primary bone marrow-derived macrophages were treated with 20 μg/mL cycloheximide, and protein samples were collected using RIPA buffer at the indicated time points. (A) Immunoblot analysis of the TNFR1 protein. The TNFR1 protein was detected using an anti-TNFR1 antibody (13377, Cell Signaling Technology). (B) TNFR1 protein levels were quantified using Image Studio Lite (Ver 5.2, LI-COR). The level at 0 h for each genotype was used as a standard for quantification. TNFR1, tumor necrosis factor (TNF) receptor type I; TRAPS, TNF receptor-associated periodic syndrome; WT, wild-type; Het, heterozygote; Hom, homozygote. [file Image_6.tif]

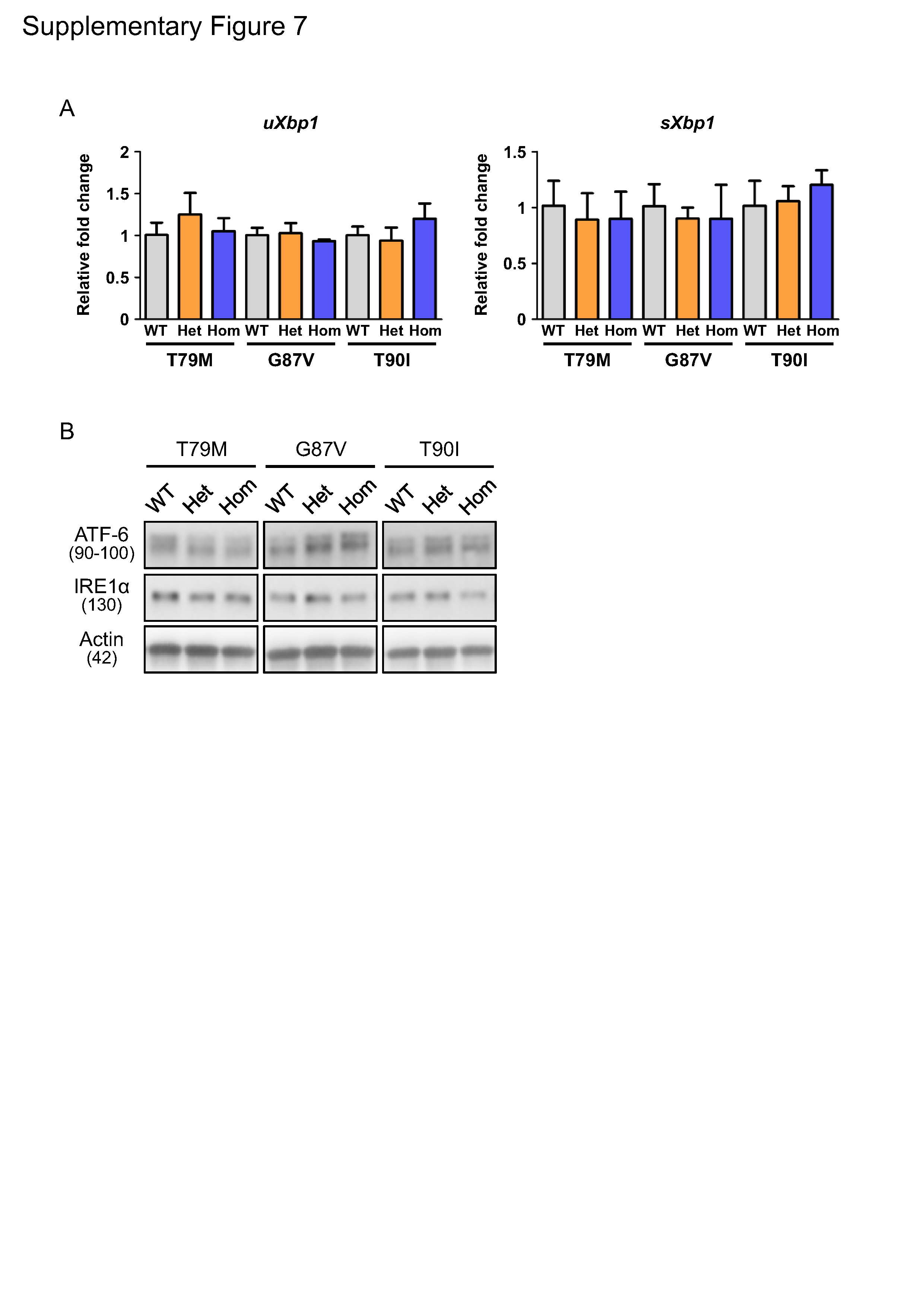

Supplement: Supplementary Figure 7 — ER stress markers in TRAPS mutant primary bone marrow-derived macrophages. Primary bone marrow-derived macrophages were cultured with M-CSF in the absence of additional stimuli. (A) Unspliced Xbp1 (uXbp1) and spliced Xbp1 (sXbp1) mRNA expression levels were determined by qPCR. The level for each WT was set at 1. Values are presented as mean ± standard deviation. (B) ATF-6 and IRE1α protein expression levels were detected by Western blot. ER, endoplasmic reticulum; WT, wild-type; Het, heterozygote; Hom, homozygote. [file Image_7.tif]
